# Improving aboveground biomass maps of tropical dry forests by integrating LiDAR, ALOS PALSAR, climate and field data

**DOI:** 10.1186/s13021-020-00151-6

**Published:** 2020-07-29

**Authors:** J. Luis Hernández-Stefanoni, Miguel Ángel Castillo-Santiago, Jean Francois Mas, Charlotte E. Wheeler, Juan Andres-Mauricio, Fernando Tun-Dzul, Stephanie P. George-Chacón, Gabriela Reyes-Palomeque, Blanca Castellanos-Basto, Raúl Vaca, Juan Manuel Dupuy

**Affiliations:** 1 Centro de Investigación Científica de Yucatán A.C. Unidad de Recursos Naturales, Calle 43 # 130. Colonia Chuburná de Hidalgo, C.P. 97200 Mérida, Yucatán Mexico; 2 El Colegio de la Frontera Sur, Laboratorio de Análisis de Información Geográfica y Estadística, Carretera Panamericana y Periférico sur s/n., San Cristóbal de las Casas, CP 29290 Chiapas, Mexico; 3 Centro de Investigaciones en Geografía Ambiental, Universidad Nacional Autónoma de México, Campus Morelia, Antigua Carretera a Pátzcuaro 8701, Col. Ex-Hacienda de San José de La Huerta, C.P. 58190 Morelia, Mexico; 4 University of Edinburgh, School of GeoSciences, Edinburgh, EH9 3FF UK; 5 CONACYT - Consorcio de Investigación, Innovación y Desarrollo para las Zonas Áridas (CIIDZA), El Colegio de San Luis (COLSAN), Parque de Macul 155, Fracc. Colinas del Parque, San Luis Potosí, S.L.P, Mexico

**Keywords:** Climatic water deficit, Forest biomass, L-band SAR, Random forest, Texture analysis, Yucatan peninsula

## Abstract

**Background:**

Reliable information about the spatial distribution of aboveground biomass (AGB) in tropical forests is fundamental for climate change mitigation and for maintaining carbon stocks. Recent AGB maps at continental and national scales have shown large uncertainties, particularly in tropical areas with high AGB values. Errors in AGB maps are linked to the quality of plot data used to calibrate remote sensing products, and the ability of radar data to map high AGB forest. Here we suggest an approach to improve the accuracy of AGB maps and test this approach with a case study of the tropical forests of the Yucatan peninsula, where the accuracy of AGB mapping is lower than other forest types in Mexico. To reduce the errors in field data, National Forest Inventory (NFI) plots were corrected to consider small trees. Temporal differences between NFI plots and imagery acquisition were addressed by considering biomass changes over time. To overcome issues related to saturation of radar backscatter, we incorporate radar texture metrics and climate data to improve the accuracy of AGB maps. Finally, we increased the number of sampling plots using biomass estimates derived from LiDAR data to assess if increasing sample size could improve the accuracy of AGB estimates.

**Results:**

Correcting NFI plot data for both small trees and temporal differences between field and remotely sensed measurements reduced the relative error of biomass estimates by 12.2%. Using a machine learning algorithm, Random Forest, with corrected field plot data, backscatter and surface texture from the L-band synthetic aperture radar (PALSAR) installed on the on the Advanced Land Observing Satellite-1 (ALOS), and climatic water deficit data improved the accuracy of the maps obtained in this study as compared to previous studies (R^2^ = 0.44 vs R^2^ = 0.32). However, using sample plots derived from LiDAR data to increase sample size did not improve accuracy of AGB maps (R^2^ = 0.26).

**Conclusions:**

This study reveals that the suggested approach has the potential to improve AGB maps of tropical dry forests and shows predictors of AGB that should be considered in future studies. Our results highlight the importance of using ecological knowledge to correct errors associated with both the plot-level biomass estimates and the mismatch between field and remotely sensed data.

## Background

Tropical forests are a significant reservoir of carbon within terrestrial ecosystems, helping to mitigate climate change and providing numerous valuable ecosystem services [1, 2]. Tropical dry forests (TDF) specifically, are the largest land cover type in the tropics [3] containing over 18% of the carbon stocks found in all tropical forests [4]. Tropical dry forests are particularly widespread in Mexico, which is home to 38% of Neotropical TDF [3]. However, TDF also experience higher rates of forest loss compared to the humid tropics due to higher population densities. Therefore, understanding the spatial distribution of aboveground biomass (AGB) and the associated carbon stock of TDF is essential to help maintain these stocks and mitigate climate change. Recent studies have produced maps of AGB or carbon density at continental and national scales [5–11]. However, such maps often have large uncertainties, particularly in tropical areas with complex vegetation structure and high AGB values [8, 9]. Within Mexico, studies by Rodriguez-Veiga et al. [8] and Cartus et al. [10] mapped AGB, reporting higher relative errors in the tropical dry forests of the Yucatan peninsula compared to other forest types in Mexico.

A commonly used approach to map the spatial distribution of AGB or carbon density is by combining forest plot data with remotely sensed information [12]. In many cases, field data is collected by national forest inventories (NFIs), which provide extensive and detailed information of vegetation attributes [13]. However, NFI plot networks can contain errors, one of them is related to the minimum diameter at breast height (DBH) used. Thus, field estimates of AGB may have errors, particularly for some vegetation types and young secondary forests [14]. Mexican NFI only measured trees > 7.5 cm DBH [15]. Excluding small trees from inventories is particularly important in TDF, as a large proportion of trees are small [16]. Small stems (< 10 cm DBH) represent between 15 and 40% of AGB in mature TDF of Mexico [17, 18], and up to 80% of AGB in young secondary TDF [17]. As the majority of the vegetation in the Yucatan peninsula consists of tropical dry secondary forest [17], NFI plots could be vastly underestimating the AGB of these forests. Another limitation of NFIs is the time involved in collecting the plot data. The last complete Mexican NFI was conducted between 2009 and 2014, within this 6-year time interval significant changes in AGB could occur, mainly by tree growth, recruitment and mortality processes. Poorter et al. [19] estimated that, after 20 years of secondary succession, Neotropical forests recover between 30 and 60% of old-growth forest AGB values. Therefore, a 6-year time interval could result in an error in AGB of 10 to 20%. Such changes in AGB are particularly challenging for remote sensing as selecting data from a single date within this six-year time interval does not take into account changes in biomass. Data obtained from chronosequences can help build predictive models of biomass changes over time [20], which could be used to correct for AGB values of NFI plots. However, whilst these sources of error have been recognized [8, 10], very few studies mapping AGB or carbon density have actually addressed it [21, 22].

In addition, different sample sizes of forest plots within different forest types, can affect the accuracy of AGB predictions in some areas. For example, deciduous forests in the Mexican NFI have a quarter the number of samples compared to semi-deciduous and semi-evergreen forests, despite covering a similar area. As a complementary approach to field inventories, LiDAR (Light Detection and Ranging) data can offer information about the vegetation structure, similar to and almost as accurately as field plots [23]. LiDAR is able to penetrate the forest canopy [24], producing a three-dimensional cloud of points of the forest structure, which can estimate AGB accurately [25]. However, due to the high costs of LiDAR data acquisition, wall-to-wall LiDAR coverage for many NFI programs is not possible, particularly in developing countries. Therefore, an alternative approach for mapping AGB with LiDAR is through the application of a two-stage upscaling method, whereby AGB from field plots is related to LiDAR data to estimate AGB along LiDAR transects. Then, the AGB of plots extracted from AGB LIDAR maps is related to satellite imagery and/or environmental information covering the entire area of interest [23, 26, 27]. This method has been shown to improve the accuracy of estimation for several vegetation structure parameters in diverse forest systems [26, 27], but, to our knowledge, has not been evaluated in TDF.

Synthetic Aperture Radar (SAR) data has also been used to successfully map AGB. Moderate wavelength SAR instruments such as the Advanced Land Observing Satellite (ALOS) Phased Array L-band Synthetic Aperture Radar (PALSAR) instrument from the Japanese Aerospace Exploration Agency (JAXA), which has a wavelength of 15 to 30 cm, can penetrate the forest canopy interacting with stems and branches, where the majority of biomass is stored [28]. The intensity of the radar backscatter signal is then related to AGB. However, mapping forest AGB using L-band SAR data does have some limitations. The relationships between radar backscatter intensity and AGB can saturate [28], typically at around 150 Mg ha-1 [29, 30], depending on vegetation type, complexity of canopy structure, or topography. The AGB in some sites of the Yucatan peninsula can exceed 320 Mg ha-1, therefore, solely using L-band SAR backscatter to map AGB would likely lead to underestimation in this region.

Several methods have been tested to overcome saturation problems, such as using SAR polarization ratios to identify the contribution of the volume of scattering from different polarizations [31–33]. Alternatively, remotely sensed information related to the vertical and horizontal structure of vegetation can be used for estimating AGB [34]. As with LiDAR data [24, 25], L-band SAR is sensitive to the vertical structure of vegetation due to its ability to penetrate through the forest. Tropical forests have a heterogeneous structure with forest canopy, canopy openings, and spacing between trees. Furthermore, forests exhibit landscape-scale heterogeneity due to land use changes that create a mosaic of forest patches of different ages. Such variations in horizontal vegetation structure have been characterized using the texture of very high-resolution imagery [35, 36]. Although the resolution of ALOS PALSAR imagery cannot discriminate individual trees and canopy openings, it can capture broader scale variation in the horizontal structure of vegetation related to the presence of forest patches with different successional age and hence forest structure [37–39]. However, the use of SAR texture data to help map AGB in TDF is still poorly understood.

Several studies have found that biomass is affected by climatic variables. Variation in temperatures and precipitation have been shown to influence forest biomass [4], as well as variation in water availability, measured as the difference between precipitation and evapotranspiration [40]. Water availability influences plant growth; sites with high water supply have higher rates of growth and recruitment, leading to high AGB. Therefore, variation in water availability influences the spatial distribution of forest biomass [19, 40]. Water availability is particularly important in seasonally dry forests, which experience severe climatic water deficit (CWD) during the dry season, which limits recruitment and tree growth [41]. Thus, including CWD data may improve the accuracy of biomass maps in TDF.

Here we address the sources of error in field and remotely sensed data and account for water availability to improve the accuracy of AGB estimates in the tropical dry forests of the Yucatan peninsula. We aim to: (1) evaluate the effect of correcting NFI plots by estimating the contribution of small trees to AGB and by taking into account biomass dynamics to better match the timing between field and remotely sensed measurements. We expect that these corrections will improve the accuracy of AGB estimates. (2) to evaluate the accuracy of biomass maps using three modelling approaches: a field plots approach, which corrects the values of AGB in NFI plots; a LiDAR plots approach, which increases sample size by estimating AGB values from a two-stage upscaling method (from field data to LiDAR transects and then from Lidar AGB maps to the whole study area); and finally a combination of these two approaches (field and LiDAR plots approach). We expect that this combined approach will yield the best estimates of AGB, since they can capture the range of forest structure and effectively increase of the sample size. Finally, (3) compare AGB map obtained in this study with existing biomass maps in Yucatan peninsula. We expect that our map will perform better and show lower estimation errors compared to previous maps.

## Methods

### Study area

**Fig. 1:**
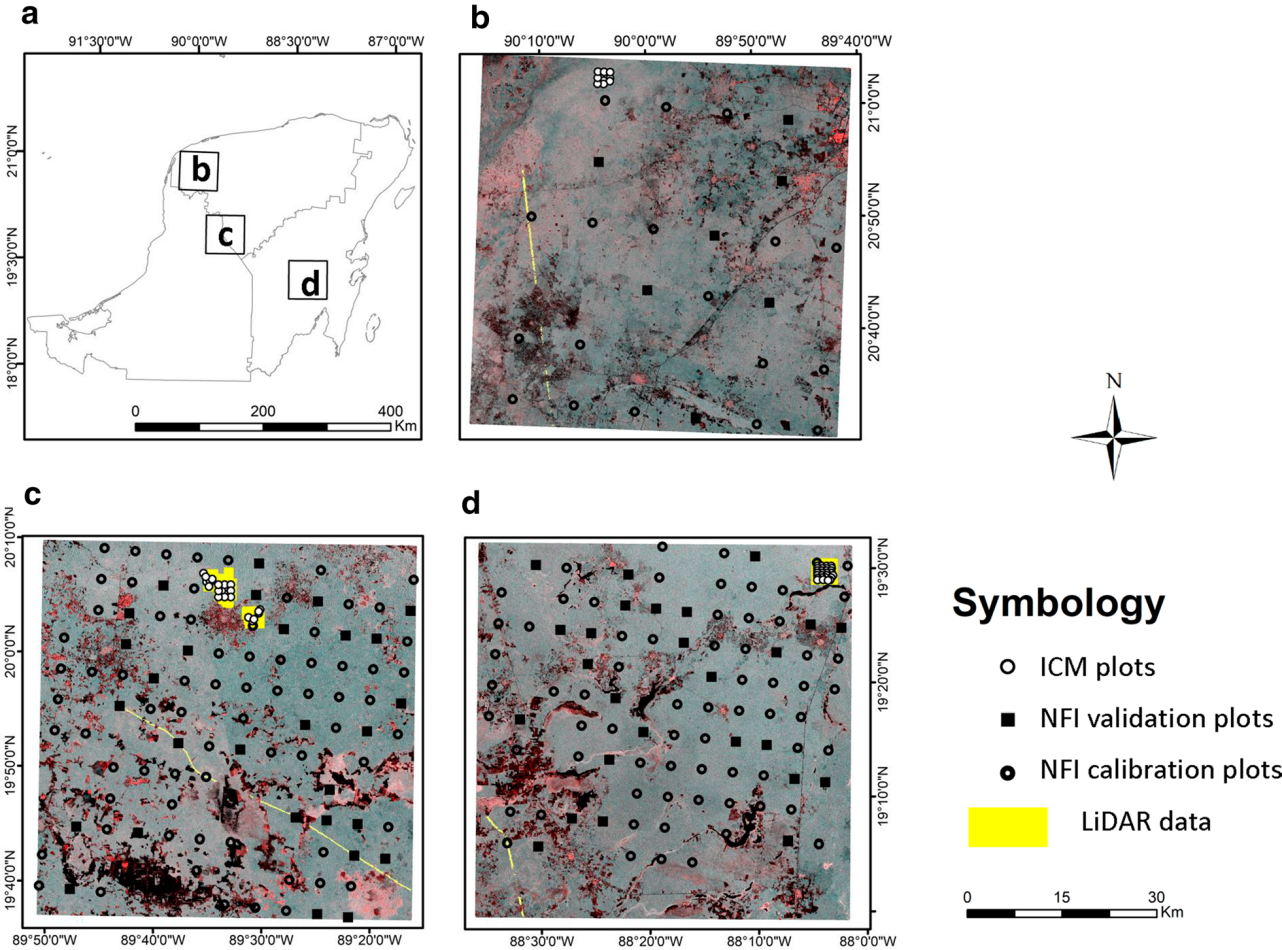
Location of the study area showing the three sites in the forest ecosystems of the Yucatan peninsula. **a** The spatial distribution of Intensive Carbon Monitoring (ICM) and National Forest Inventory (NFI) field plots as well as LiDAR data for each site of tropical dry forest: deciduous (**b**), semi-deciduous (**c**) and semi-evergreen (**d**)

The study was conducted in three sites of 3600 Km^2^ each, which cover the full environmental gradient of the most important TDF ecosystems of the Yucatan Peninsula, Mexico: deciduous, semi-deciduous and semi-evergreen (Fig. 1). The climate in the peninsula is tropical warm with a dry season from November to April and a fairly flat topography [42]. The mean annual temperature is 27°, 26° and 25 °C with a mean annual precipitation range of (800–900, 1000–1100, 1000–1300) mm year^−1^ for the deciduous, semi-deciduous and semi-evergreen TDF, respectively. Deciduous forests have the lowest canopy height, while the semi-evergreen forest have the tallest canopy height with a more complex vegetation structure than the other sites [12, 43].

### Field data

We used two different data sets of field plots for calculating aboveground biomass, National Forest Inventory (NFI) plots sampled between 2009 and 2014 and Intensive Carbon Monitoring (ICM) plots sampled in 2014 and 2015. Each NFI sampling unit consists of 4 circular 400 m^2^ plots within an area of 1 ha, in which all trees > 7.5 cm diameter at breast height (DBH, 1.3 m) were identified and measured. In total, 232 sampling units were established and inventoried in the study area, on a fixed grid of 5 × 5 km in the semi-deciduous and semi-evergreen forests, and 10 × 10 km in the deciduous forest [15]. The ICM plots have a similar design to that of the NFI, with the addition of a nested 80 m^2^ subplot where all trees with DBH between 2.5 and 7.5 cm were sampled. A total 80 ICM sample units were measured in the study area using a systematic sampling design [12] (Fig. 1). All plants inside each sampling unit were identified at species level, and several vegetation attributes were measured, including DBH, and height. In both data sets, those sampling units where at least one plot was deforested or converted to another land use by 2015 were discarded from the analyses (n = 14 from NFI plots, n = 11 from ICM plots); we used the remaining 287 plots (n = 218 from NFI plots, n = 69 from ICM plots) for AGB mapping and validation.

We used local and regional allometric equations to calculate aboveground biomass (AGB) of tropical dry forests (TDF). The equations take into consideration the vegetation type as well as DBH, height and wood density of trees (Table 1). For most of the tree species sampled, we obtained wood density values from local studies, while for some tree species these values were obtained from the literature (see Additional file 1: Table S1). For those species without wood density values, we assigned the average of the values at the genus level or for a sampling unit. All plot-level AGB values were transformed to standard units (Mg ha^−1^).

**Table 1:** Description of allometric equations used to estimate aboveground biomass for field plots

Author of equation	Type of forest	Biological form/class size	Allometric equation
Ramírez et al. [44]	Deciduos and semi-deciduos	Tree/DBH < 10 cm	EXP(− 4.1392 + 0.99 * LN(DBH^2^ * LENG) + 1.2268 * DENS)
Chave et al. [45]	Deciduos and semi-deciduos	Tree/DBH ≥ 10 cm	DENSI * EXP(− 0.667 + 1.784 * LN(DBH) + 0.207 * LN(DBH)^2^ − 0.0281 * LN(DBH)^3^)
Guyot [46]	Semi-evergreen	Tree/DBH < 10 cm	EXP(1.3636 * LN(DBH) + 1.615 * LN(LENG) − 2.9267)
Cairns modified [47] by Urquiza-Haas et al. [48]	Semi-evergreen	Tree/DBH ≥ 10 cm	EXP(− 2.12605 + 0.868 * LN(DBH^2^ * TH) + (0.0939/2)) * (DENS/0.7)
Chave et al. [49]	Deciduos, semi-deciduos and semi-evergreen	Liana/DBH ≥ 2.5 cm	EXP(0.049 + 2.053 * LN(DBH))
Frangi and Lugo [50]	Deciduos, semi-deciduos and semi-evergreen	Palms/DBH ≥ 10 cm	− 4.51 + (7.7 * LENG)

As small trees (< 7.5 cm DBH) were not measured in NFI plots, we used ICM plots to calculate an AGB correction factor for NFI plots in each forest type (hereafter small tree correction). The proportion of small trees varies with successional age, which is also strongly related to AGB in TDF [18]. Since stand age was not measured in NFI plots, we used AGB as a proxy for forest stand age and stratified both NFI and ICM plots in 5 strata using 10, 25, 50 and 75% percentiles of AGB. Then, we calculated a correction factor in each stratum using the AGB calculated for small trees in the ICM plots. The correction factor for each class and type of TDF are shown in Additional file 2: Table S2.

Since NFI plots were measured over a 6-year time interval (2009–2014) we also corrected NFI plots by stand age (hereafter age corrected), considering the AGB vs stand age functions obtained from two chronsequences: one for the deciduous and semi-deciduous forests [51] and the other for semi-evergreen forests [52] (see Additional file 3 Fig. S1). We corrected NFI plots to have a baseline year of 2015. First an approximate age for each sampling unit was estimated using the inverse of the AGB vs age function. Then, the difference between 2015 and the year of the field measure was added to the estimated age. Finally, AGB was calculated using the AGB vs stand age function for the updated age.

Finally, to achieve objective 1 (to evaluate the effect of correcting NFI plots using ecological information), we used exclusively the NFI plots (218): 70% (152 plots) were utilized to calibrate the models, while the 30% (66 plots) were used for validation. For objective 2 (to evaluate if increasing sample size by using biomass estimated with LiDAR plots could increase the accuracy of AGB estimates), we used both NFI and ICM plots (287). We used 200 of these plots (70%) to calibrate the models while the remaining 87 plots (30%) were used to validate the models. The plots used for validation were selected in the same proportion considering the three different TDF types and the 5 strata of AGB values.

### LiDAR data

Lidar data were collected under two different canopy conditions: leaf–on and leaf-off by a private contractor and NASA G-LiHT airborne imager [53] respectively, during 2014 (Fig. 1). Both datasets were collected with the same LiDAR sensor, with similar settings and flight parameters, see [54]. Both data sets had the same pulse density (> 5 pulses per m^2^). The accuracy of predictions of AGB, in this area, is not significantly influenced by using leaf-on or leaf-off LIDAR data, with differences in the relative RMSE of less than 2% [54].

LiDAR data were normalized with a 1 m^2^ resolution digital terrain model to eliminate the elevation of the ground from the height of returns. The LiDAR metrics were calculated at 1 m^2^ resolution. See Table 2 for a description of the LiDAR explanatory variables. We used the FUSION software for processing LiDAR data [55].

**Table 2:** Description of explanatory variables used to estimate above ground biomass

Type of variable	Variable	Description
LiDAR	Height metrics	These metrics includes mean, median, mode, maximum and minimum of canopy height, the variations of canopy height (variance, coefficient of variation) as well as percentiles 1, 5, 10…100 and L-moments. See [49] for description and formulas.
	Point density metrics	Metrics used to evaluate canopy coverage. See [49] for description and formulas.
ALOS PALSAR	HH	Radar backscatter HH polarization
	HV	Radar backscatter HV polarization
	NDBI	The normalized difference backscatter index between the HH and HV bands. [32].
	Texture of HH, HV and NDBI	The second-order texture measures used in this study are homogeneity (hom), contrast (cont), dissimilarity (dis), entropy (ent), angular second moment (asm), mean (mean), variance (var), and correlation (cor). See Haralick et al. [50] for details and formulas.
Climate	CWD	The Climatic Water Deficit (CWD), calculated as the difference between rainfall and evapotranspiration in the dry months [51].

### SAR data

Six ALOS PALSAR-2 (Advanced Land Observing Satellite Phased Array L-band Synthetic Aperture Radar) mosaic tiles with 25 m resolution covering the study area during 2015 were obtained from the Japanese Aerospace Exploration Agency. The 4 orbits covering the 6 mosaic tiles were acquired form September 11 and November 18 during the rainy season. The PALSAR-2 mosaic data has undergone pre-processing, which includes; ortho-rectification, slope correction and radiometric calibration for both polarizations: HH and HV [56]. The digital number in these mosaics were converted into backscatter coefficients (γ°) using the following equation [57]:

1 $$ \gamma^\circ \left( {dB} \right) = 10\log_{10} \left( {DN^{2} } \right) - 83.0 $$

where DN is the digital number expressed as unsigned short integer. To reduce the speckle noise without sacrificing image structure, we applied 3 × 3 pixel LEE filter [58] to ALOS-PALSAR-2 backscatter images.

We also calculated the normalized difference backscatter index (NDBI) between the HH and HV backscatter coefficients with the equation:

2 $$ NDBI = \frac{HH - HV}{HH+HV} $$

This ratio index helps to differentiate vegetation types due to the different contribution of volume scattering in different polarizations [30–32].

We used SAR texture analysis to produce more information that could be related to AGB estimation. Texture analysis quantifies the variability in backscatter values of neighboring pixels [59]. We calculated eight second-order texture measures for the two backscatter polarizations (HH and HV), and for the NDBI ratio index using ‘glcm’ pack of R software [60]. Since most of the Yucatan peninsula is on flat terrain, an averaged texture value was obtained from the values for four directions (0°, 45°, 90°, 135°). The texture measures employed in this study are shown in Table 2. A window size of 3 × 3 pixels was used to calculate the second-order texture measures, because the resulting area is the closest to the field plot size (1 ha). We extracted 24 variables considering 8 texture measures and three bands that were used in a random forest model to relate with biomass field data.

### Climate data

A total of 497 climatic stations covering the Yucatan peninsula and two adjacent States (Chiapas and Tabasco) were used to obtain a continuous surface of temperature, rainfall and evapotranspiration using spatially interpolated values through the kriging method (Additional file 4 Fig S2 (b)). Climate data were obtained from 1920 to 2012 from the Climate Computed Project [61]. After a quality control of the meteorological stations, we removed 72 stations that did not contain the full 12-month data or did not have the complete series of data in the interval of 1920 to 2012. In total we used 425 stations for modeling, presenting a homogeneous distribution throughout the study area, with adequate spatial coverage.

Raster data of rainfall and evapotranspiration in the 12 months covering only the Yucatan peninsula were used to calculate the climatic water deficit (CWD) map (see Additional file 4: Fig S2 (a)). This index measures the deficit of water in the dry months and is calculated as the difference between rainfall and evapotranspiration during these months, in this case from February to May [19, 62]:

3 $$ CWD_{\left( i \right)} = \mathop \sum \nolimits_{i = 1}^{i = 12} Min \left( {0, P_{i} - ET_{i} } \right) $$

where *P*_*i*_ is the monthly rainfall, ETi monthly potential evapotranspiration and i is the month. The *ET*_*i*_ was estimated from the Priestley-Taylor equation [63] one of the most commonly used to calculate potential evapotranspiration at wide spatial scales, which incorporates temperature, latitude and solar net radiation and was calculated using the ‘EcoHydRology’ package of R software [60].

The CDW measures drought condition and, by definition, has negative values. This means that places with 0 values do not have a water deficit, while areas with high water stress have negative values of the index. In addition, CWD is highly correlated with AGB, because when there are lower (more negative) values of CWD, there is less water availability, resulting in lower forest biomass growth [19].

### Estimation and mapping of AGB from LiDAR data

We carried out a regression analysis between AGB and LiDAR metrics using 69 NFI and ICM field plots that fell within the LiDAR transects, using a subset regression procedure with ‘leaps’ pack of R software [60]. The response variable (AGB) was square-root transformed to meet linearity assumptions [64], and the independent variables were the mean LiDAR metrics values of 1 m^2^ for each sampling plot. The validation of the models was evaluated by the leave-one-out cross-procedure [65]. The predicted and observed values of AGB were compared using the coefficient of determination (R^2^), the root mean square error (RMSE), the relative root mean square error (%RMSE) calculated as the RMSE divided by mean observed values of AGB and the bias was calculated as the average values of errors (difference between predicted and observed AGB values).

In addition, we mapped AGB in areas covered with LiDAR data using a map band function based on the fitted regression equation as well as the layers of the LiDAR metrics included in the model using 1 m^2^ pixel resolution. Then, AGB LIDAR values were extracted as means values of estimated biomass inside a circular plot of 1 ha. In total we have 5021 plots that filled the area covered by LiDAR data, which covered only 0.46% of total study area.

### AGB model development and validation

We built random forest models to estimate AGB using backscatter and texture variables from ALOS PALSAR, as well as CWD. The number of decision trees was set to 500 and we determined the optimal number of predictor variables to retain at each node for each model, using the ‘ModelMap’ package in R [66].

To evaluate the effect of small tree and stand age corrections on the accuracy of AGB estimates, we built the following four random forest models to estimate AGB using: (1) NFI plots (small tree corrected); (2) NFI plots (age corrected); (3) NFI plots corrected by both factors; (4) uncorrected NFI plots. Approximately 70% of the data (152 plots) were selected using a stratified random design and were used to fit the models. The remaining 30% of data (66 plots) were used to test model performance. The accuracy of the estimated forest AGB from each model was evaluated by directly comparing the estimated result with an independent set of data of ground inventory plots (66 plots). We used R^2^, RMSE,  %RMSE, bias and calculated standard deviation of errors to compare the predicted and observed values of AGB. Additionally, a spatial autocorrelation test was applied on residuals of calibrated models using Moran’s I test.

To evaluate the effects of using AGB estimates from LiDAR and/or field plots, we used three model approaches. The first approach (field plots approach) used AGB from NFI (corrected values) and ICM plots to calibrate the model. The second approach (LiDAR plots approach) used estimated AGB values from LiDAR maps, we applied a two-stage upscaling method; from field plots to LiDAR strips and then from LiDAR AGB maps to the entire study area. The third approach (field and LiDAR plots approach) combined both sources of AGB values: field plots and LiDAR plots. In the three models, the validation plots from the field plots approach (87 plots) were used as an independent data set to validate the models performance using R^2^, RMSE,  %RMSE and bias. This set of data was chosen to preserve the overall distribution of AGB values. Seventy percent of the sampling field plots (200 plots) were used to fit the model in the approach that used field plots. In the case of the LiDAR plots approach, we used 5021 AGB sample units, obtained from AGB maps derived from LiDAR strips, to calibrate the model. Finally, the approach that combined both sets of data, had 5221 sample units to fit the model.

Maps with the spatial distribution of AGB and coefficient of variation of AGB estimates in the study area, were created with the random forest model using the ‘ModelMap’ pack of R software. The random forest model was performed considering the mean of all the trees of the response variable, in this case AGB. Therefore, these individual tree predictions can also be used to map measures of uncertainty such as coefficient of variation maps (dividing the standard deviation by the mean). These maps provide a visualization of spatial regions of higher uncertainty.

### Comparison of mapped AGB with other studies

The mapped AGB values from the best model in this study was compared to previous maps of AGB or carbon density maps previously converted to biomass [8, 10]. These studies both used the NFI plots to produce an AGB or carbon density for Mexico, however we only compared the results for the tropical dry forests of the study area. The study of Cartus [10] mapped AGB carbon density using random forest and three groups of variables: canopy density estimates from Landsat, backscatter from ALOS PALSAR and elevation derived from shuttle radar topography mission (STRM). The study of Rodriguez-Veiga [8] estimated AGB with the maximum entropy algorithm using several explanatory variables: vegetation indices derived from MODIS, backscatter from ALOS PALSAR and elevation obtained from SRTM. Neither previous study corrected AGB values from NFI plots for small trees and stand age (biomass dynamics). We compared the AGB maps in the three studies with the AGB estimated in validation plots (87 plots) using R^2^, RMSE,  %RMSE and bias. In addition, we calculated the distribution of AGB maps in the 3 studies and we also obtained the mean values and 95% confidence intervals of the differences between reference and predicted AGB values stratified by reference AGB ranges.

## Results

### AGB estimated in LiDAR transect data

**Fig. 2:**
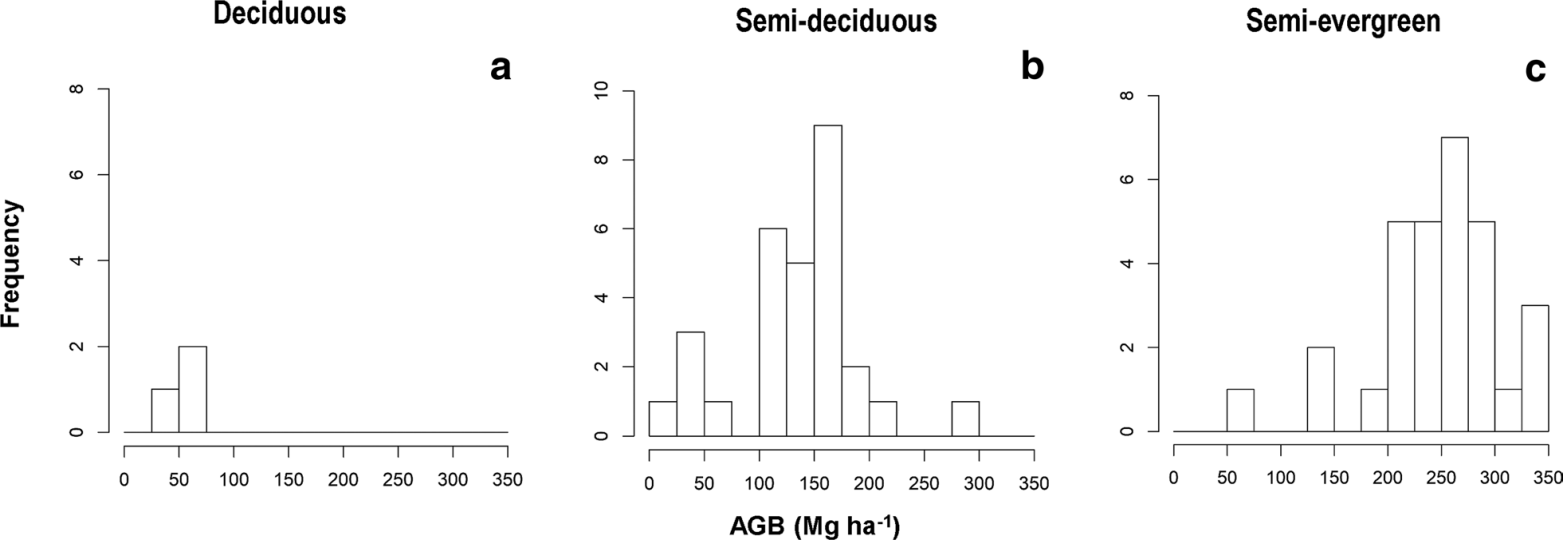
Frequency histograms of AGB from field plots within LiDAR data for each tropical dry forest type: deciduous (**a**), semi-deciduous (**b**) and semi-evergreen (**c**)

To estimate AGB along the LiDAR transects, we have 69 plots from NFI and ICM that were located within the LiDAR data, the frequency distribution of AGB values of these plots for each forest type can be seen in Fig. 2. Results of linear regression analysis showed a high association between AGB and LiDAR data with an R^2^ = 0.87 (Additional file 5: Table S3). The cross-validation results showed that the AGB estimation based on LiDAR data is accurate with a high R^2^ = 0.85 and low RMSE = 34.8 Mg ha^−1^. In addition, the relative RMSE has a value of 19.7% and a bias close to 0 (− 1.1) (Additional file 6: Fig. S3). Therefore, values of AGB estimated from LiDAR data could be suitable for calibration in models used to estimate AGB from ALOS PALSAR and CWD for the study area.

### Effects of small trees and stand age on the accuracy of AGB estimates

**Fig. 3:**
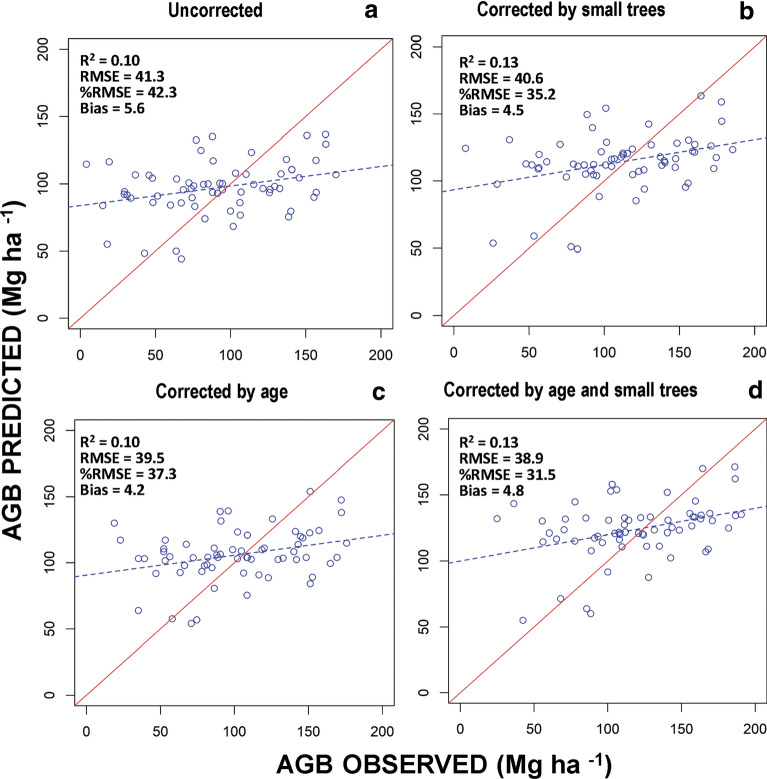
Model validation showing observed versus predicted AGB (Mg ha^−1^): uncorrected AGB values of NFI plots (**a**), AGB values of NFI plots corrected for small trees (**b**), AGB values of NFI plots corrected for stand age (**c**) and AGB values of NFI plots corrected for both small trees and stand age (**d**)

The random forest models used to estimate AGB from three sets of explanatory variables (backscatter and texture from ALOS PALSAR as well as CWD), indicate moderate percentage of AGB variance explained by the models in the calibration data (R^2^ values are from 0.17 to 0.19), and validation data (R^2^ values are from 0.10 to 0.13) (Table 3). We found no significant spatial autocorrelation (p > 0.05) of residuals for any of the four models. The performance of the models in the validation procedure indicated that R^2^ values increased and the error decreased as each correction factor was applied. The relative RMSE decreased by 8.4 and 6.2% when the correction for small trees and stand age were applied respectively (Table 3, Fig. 3). This result indicates that the correction for small trees performed better than the correction by stand age. However, when both types of corrections are applied to the AGB estimates from the NFI, the relative RMSE decreases by 12.2%. The bias in all groups of corrected and uncorrected plots were close to 0 (from 0.2 to 5.6), which explains why RMSE and SD of error have very similar values, since RMSE^2^ = bias^2^ + SD^2^.

**Table 3:** Evaluation statistics for predicting aboveground biomass from ALOS PALSAR and climate variables, using corrected and uncorrected NFI plot data

Data	NFI plots	R^2^	RMSE	%RMSE	Bias	SD of error
Calibration (n = 152)	Uncorrected	0.17	44.7	48.1	− 0.3	44.3
	Corrected by small trees	0.18	41.1	37.4	0.5	44.9
	Corrected by age	0.19	44.8	43.7	0.2	41.2
	Corrected by age and small trees	0.18	42.0	35.4	0.2	42.2
Validation (n = 66)	Uncorrected	0.10	41.3	45.0	5.6	40.8
	Corrected by small trees	0.13	40.1	36.6	4.5	39.8
	Corrected by age	0.10	39.5	38.8	4.2	39.2
	Corrected by age and small trees	0.13	38.9	32.8	4.9	38.6

### Effects of using AGB estimates from LiDAR and/or field plots

**Fig. 4:**
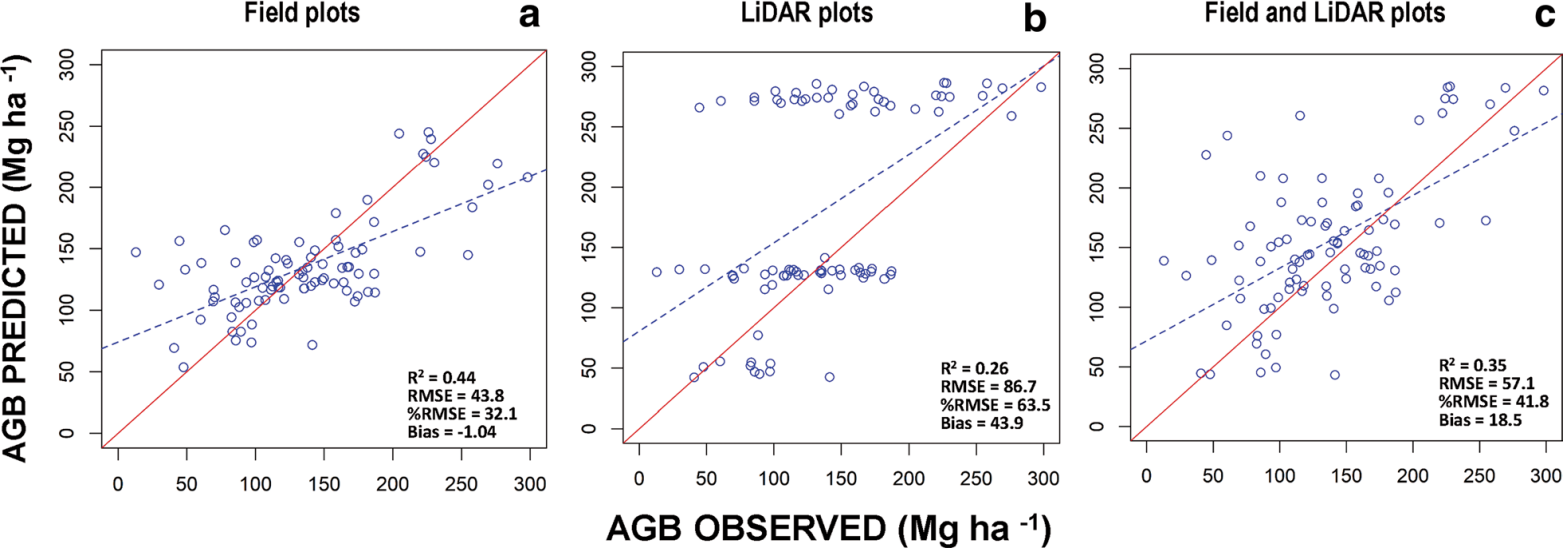
Model validation showing observed versus predicted AGB (Mg ha^−1^): AGB calculated from field data (ICM and NFI plots) (**a**), AGB estimated from LiDAR plots (**b**) and AGB obtained from both field data and LiDAR plots. **c** Red lines show 1:1 reference lines and dashed lines show regression lines

The random forest model estimated AGB from three sets of explanatory variables (backscatter and texture from ALOS PALSAR as well as CWD) indicating moderate to high agreement between observed and predicted values for the calibration data (R^2^ values from 0.52 to 0.84), and validation data (R^2^ values from 0.26 to 0.44). There was no significant spatial autocorrelation (p > 0.05) for the residuals of the three models. For the calibration data the R^2^ values had higher correspondence between observed and predicted data and lower errors for the modelling approaches that used LiDAR plots alone or Lidar and field plots combined (LiDAR only; R^2^ = 0.84,  %RMSE = 20.3, bias = 0.6, LiDAR & Field plots; R^2^ = 0.83,  %RMSE = 21.4, bias = 1.2), compared to that which used only field plots (R^2^ = 0.52 and  %RMSE = 37.4, Table 4). However, validation data showed the opposite pattern: a higher correspondence between observed and predicted data and lower errors for the modelling approach that used only field plots (R^2^ = 0.44,  %RMSE = 32.1), compared to those that used the LiDAR plots alone or LiDAR and field plots combined (LiDAR only; R^2^ = 0.26,  %RMSE = 63.4, bias = 43.9, LiDAR & Field plots; R^2^ = 0.35,  %RMSE = 41.8, bias = 18.5; Table 4, Fig. 4). Thus, the accuracy of predictions of AGB is higher and with low bias values when using field plots compared to LiDAR plots. The positive high bias values using LiDAR plots indicate over estimation of biomass and explain why SD of errors are higher than RMSE.

**Table 4:** Evaluation statistics for predicting aboveground biomass from ALOS PALSAR and climate variables, using field and LiDAR biomass plots

Data	Approach	n	R^2^	RMSE	%RMSE	Bias	SD of error
Calibration	Field plots	200	0.52	49.0	37.4	1.2	49.1
	LiDAR plots	5021	0.84	31.3	20.3	0.5	31.3
	Field and LiDAR plots	5221	0.83	32.8	21.4	0.6	32.8
Validation	Field plots	87	0.44	43.8	32.1	− 1.1	43.8
	LiDAR plots	87	0.26	86.7	63.4	43.9	74.6
	Field and LiDAR plots	87	0.35	57.1	41.8	18.5	54.0

### Spatial distribution of ABG and its uncertainty

**Fig. 5:**
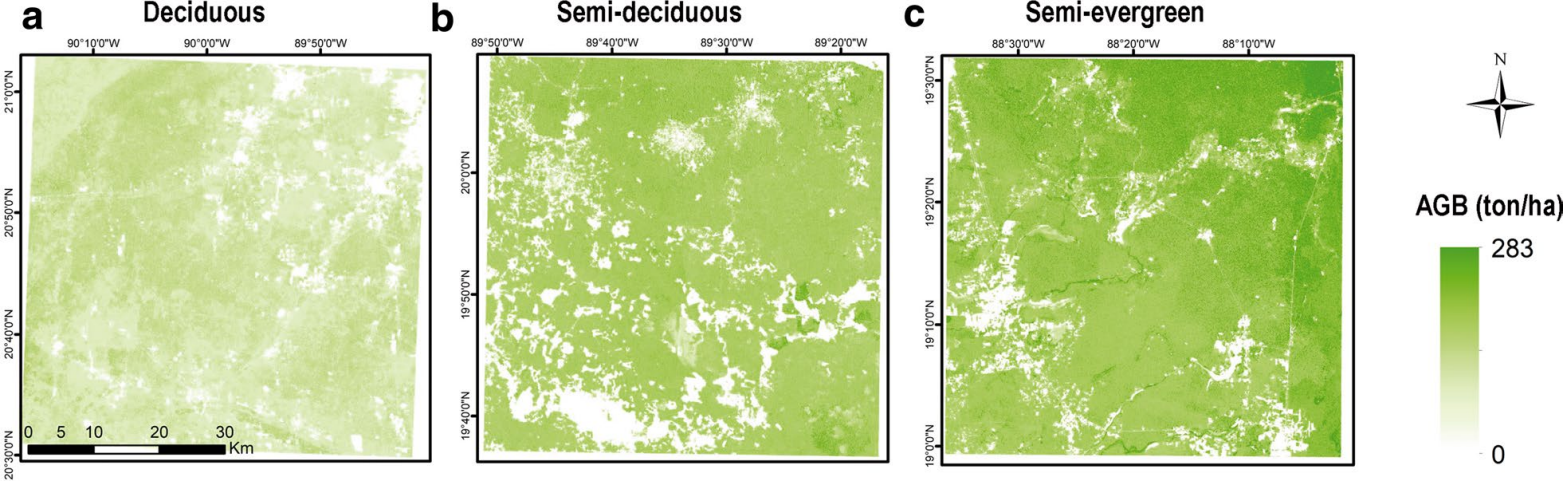
Above ground biomass maps for each tropical dry forest site: deciduous (**a**), semi-deciduous (**b**) and semi-evergreen (**c**)

**Fig. 6:**
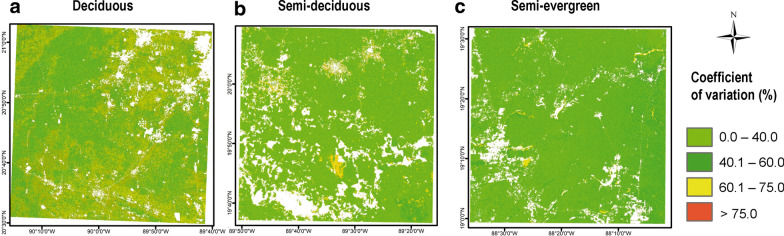
Above ground biomass uncertainty maps for each tropical dry forest site: deciduous (**a**), semi-deciduous (**b**) and semi-evergreen (**c**)

The forest AGB map of this study showed values ranging from 40 Mg ha^−1^ for deciduous TDF to 283 Mg ha^−1^ for the semi-evergreen TDF (Fig. 5). The average AGB estimated values were 99.2 Mg ha^−1^ with a standard deviation of 48.1 Mg ha^−1^. The average value for each type of TDF was 69.3, 100.4 and 127.5 Mg ha^−1^ respectively for deciduous, semi-deciduous and semi-evergreen forests. The uncertainties in most of the cases were below 40% of the CV;, however, the deciduous TDF presented higher uncertainties of up to 60% of CV (Fig. 6). These results are in agreement with the number of samples in each forest type, since the lowest number of samples corresponded to the deciduous TDF.

### Comparison of AGB maps with previous studies

**Fig. 7:**
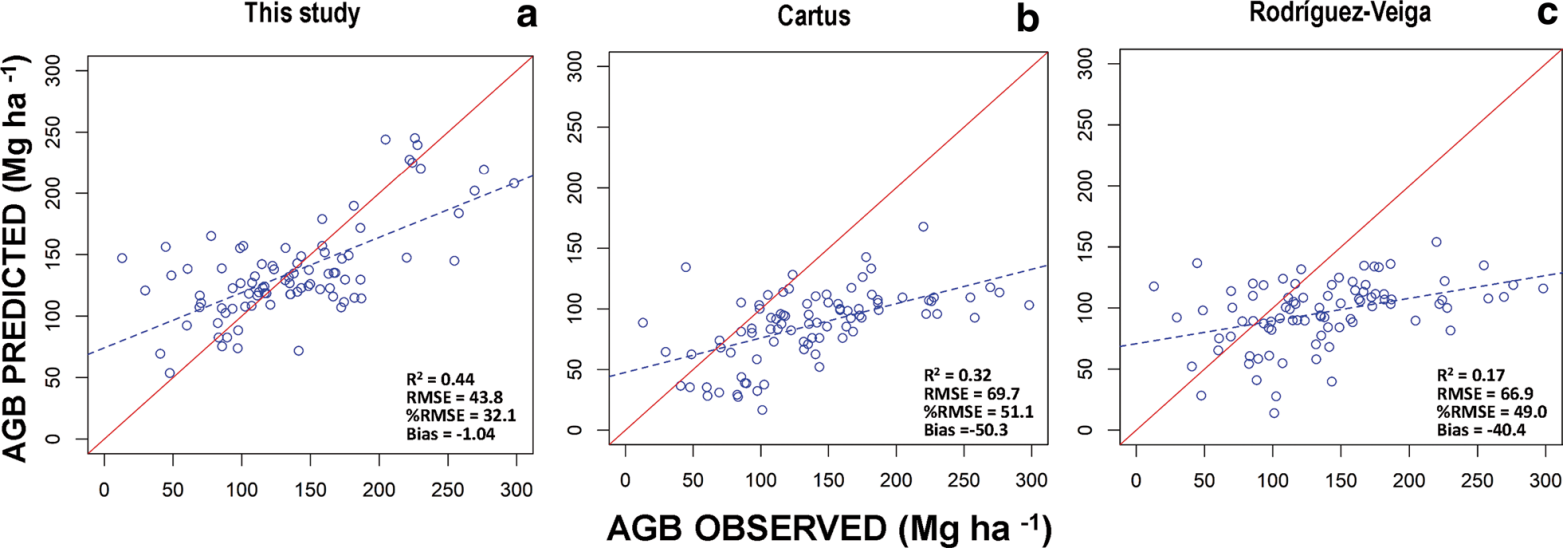
Observed versus predicted AGB (Mg ha^−1^) of validation plots in this study (**a**), in the study of Cartus et al. [9] (**b**) and in the study of Rodriguez-Veiga et al. [7] (**c**) within our three study sites. Red lines show 1:1 reference lines and dashed blue lines show regression lines

**Fig. 8:**
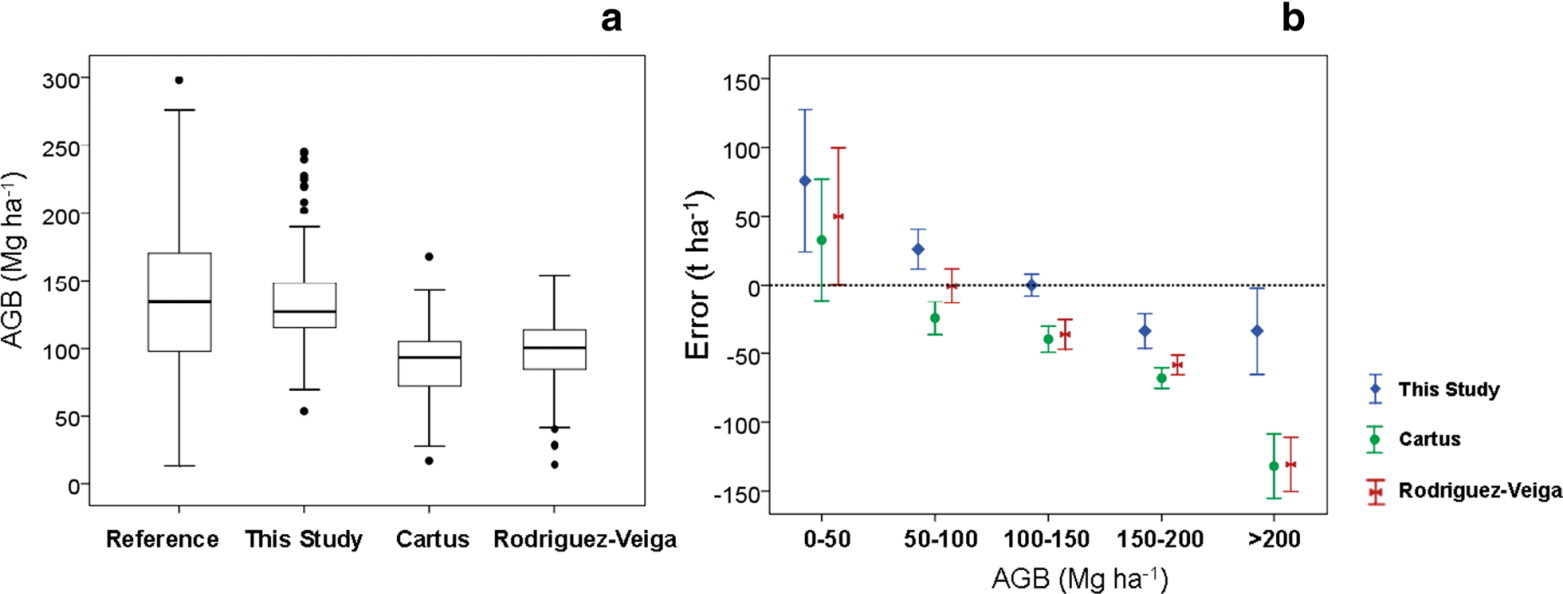
Boxplots of the reference data (used for validation) and predicted AGB in this study, and in those by Cartus et al. [9] and Rodriguez-Veiga et al. [7] (**a**). Mean values and 95% confidence intervals obtained as the differences between reference and predicted AGB values of this study, and those of Cartus et al. [9] and Rodriguez-Veiga et al. [7] and stratified by reference AGB ranges (**b**)

The validation analysis revealed that the agreement between observed and predicted values was better for this study (R^2^ = 0.44) compared to previous ones (R^2^ = 0.32 for the map of Cartus [10] and R^2^ = 0.17 for the map of Rodriguez-Veiga [8]. Similarly, the relative RMSE of this study was the lowest of the three maps (32.1% in this study, 51.1% in the study of Cartus [10] and 49.0% in that of Rodriguez-Veiga [8]) (Fig. 7). We also observed that the bias in this study was close to 0 (− 1.04) compared with the high negative bias values of − 50.3 and − 40.4 for the studies of Cartus [10] and Rodriguez-Veiga [8] respectively, indicating large under estimations of biomass in the study area. In addition, the ranges and mean AGB values differed among the three maps. The map from this study displays significantly higher values of AGB compared to the previous studies, but showed similar ranges and mean AGB values compared to the reference data from the NFI and ICM plots (Fig. 8a).

When dividing the estimates of AGB from the three maps into five categories of AGB ranges and comparing them to the reference data, we can see an overestimation of AGB in the three maps for low biomass values (< 50 Mg ha^−1^), with no significant differences among the three maps. However, our study also presented a small overestimation of AGB in biomass levels between 50 and 100 Mg ha^−1^. This graph also revealed that all studies presented under estimation of AGB for biomass values larger than 100 Mg ha^−1^. Nevertheless, the differences between reference data and estimated AGB values were lower in this study compared to the previous ones (Fig. 8b).

Finally, the maps and the frequency distribution of AGB in the three studies showed that the maps of Cartus [10] and Rodriguez-Veiga [8] have a narrow distribution of AGB values with few observations above the mean values (Additional file 7: Fig. S4).

## Discussion

To achieve the main objective of the research, improving the accuracy of AGB maps of tropical dry forests in the Yucatan peninsula, we revised the main sources of errors that can affect AGB estimation. In addition, we evaluated ways of correcting the main groups of errors: (i) in biomass estimation at the plot level, (ii) sampling error, (iii) the match between field and remote sensing measures and (iv) in AGB estimation using remote sensing and environmental data [67]. To reduce the errors for assessing biomass at the plot level, we used local allometric equations and wood density values that were measured in the study area [68]. However, another important error for estimating AGB in the plots is that NFI did not measure small trees (DBH < 7.5 cm), which account for a considerable proportion of AGB in the secondary TDF of the Yucatan peninsula [18]. To address this problem, we measured an additional set of plots (ICM plots) to correct the AGB values of the NFI plots as described in the methods. An evaluation of the effect of these corrections indicated that the relative RMSE for estimating AGB from ALOS PALSAR and climate data, decreases by 8.4% compared to the uncorrected plots.

Another common group of errors derives from mismatches between field and remotely sensed measurements; such mismatches include spatial location and the differences between the size of the sampled unit and the pixels of the imagery. However, these two errors decrease as plot size increases [12, 68]. Here we used a sample unit of 1 ha, and adjusted the grain size of the imagery accordingly, so we expect low error values. Nevertheless, another mismatch arises from temporal differences between field and remotely sensed measurements. In this study, NFI plots were measured between 2009 and 2014, while ALOS PALSAR was acquired in 2015. To solve this problem, we used predictive models of biomass changes over time, using additional data from two chronosequences [51, 52], which accounted for growth, recruitment and mortality of trees [20]. Our evaluation of the effect of taking into account biomass dynamics revealed that when this correction was used, the relative RMSE for estimating AGB from ALOS PALSAR and climate data, decreases by 6.2% compared to uncorrected data. Few previous studies have handled this error, one of them applied an approach for updating biomass values of plots using growth rates [21]. We used the chronosequence approach to estimate stand age based on AGB and then estimated temporal increases in AGB during the time elapsed between the field inventory and image acquisition. However, in this study, one of the chronosequence models was built for the semi-deciduous forest and it was applied to both deciduous and semi-deciduous forests. This may be one of the reasons for the greater uncertainty in the deciduous compared to semi-deciduous forest (Fig. 6), since the rate of AGB change across secondary succession varies between forest types and land-use history [22].

An important and novel result of this research was to correct AGB values of NFI plots for failing to measure small trees as well as temporal differences between remotely sensed data and field measurements. Considering both sources of error, our study found that the relative RMSE decreased by 12.2% compared to the uncorrected plot data. These results suggest that reducing errors at the plot-level is critical to improve the accuracy AGB maps, as well as for reducing uncertainty of these maps. Improving the accuracy of AGB estimates requires reducing the errors from the different processes involved in mapping AGB or carbon density over large areas. Most studies so far have focused on the errors due to methods of prediction and the type of remotely sensed and environmental data used to estimate biomass. Recently, field-based biomass estimation errors have gained more importance among the remote sensing community [13, 21, 29].

Another group of errors is related to differences in sampling intensity; as we pointed out earlier, deciduous forest had almost a quarter the number of samples compared to semi-deciduous and semi-evergreen forests in the Yucatan peninsula [15]. We used an approach to overcome this problem by combining both field and LiDAR plots or using LiDAR plots for training a model to predict AGB in larger areas. Contrary to our expectations, we found that the correspondence between observed and predicted values of AGB was lower and the relative error was much higher for the LiDAR plots approach (R^2^ = 0.26 and  %RMSE = 63.4), compared to the approach that used only field plots (R^2^ = 0.44 and  %RMSE = 32.1), so combining both data sets did not improve the accuracy of AGB estimation (R^2^ = 0.35 and  %RMSE = 41.8) obtained from field plots. Therefore, the evaluation using an independent set of plots showed that the field approach performed better than the other two methods. These results concur with those of Urbazaev et al. [6] who mapped AGB for all of Mexico.

Several studies have shown that using biomass estimated from LiDAR plots in a two-stage upscaling method improved the accuracy of predictions of canopy height of woodlands [69], AGB of tropical forests [70] and several vegetation-structure attributes of boreal forest [71]. Such success requires a good performance of the model that relates field AGB measurements and LiDAR metrics, together with a good representation by the LIDAR plots of the range of AGB conditions over the different vegetation types in the study area [69, 71]. In our study, the model that related AGB field measurements and LiDAR data provided high model fit and AGB estimation accuracy (R^2^ = 0.87 and 0.85, respectively) as well as low relative error (%RMSE = 19.7). Nevertheless, field plots located within the LiDAR data did not capture the range of variability of AGB in the three types of TDF (Fig. 2) and therefore AGB in LiDAR plots was overestimated (see high positive values of bias in Fig. 7). For example, there are few observations of LiDAR plots with biomass values lower than the mean AGB in the semi-evergreen TDF (Additional file 8 Fig. S5 (e)). This is because the LiDAR flights covered a small area dominated by old-growth forests with high AGB values where the ICM plots were located. In addition, the area sampled with LiDAR was very small (less than 1% of the total study area) and not representative enough. This is likely why we found a better prediction of AGB values using the field plots approach, since field plots covered a much larger and more representative area. Consequently, a recommendation for making LiDAR plots representative of the area of interest is to acquire at least 6% of the study area in a random sampling strategy [69]. Another alternative is applying a stratified sampling design for data acquisition, which will reduce the area sampled but will requires a priori knowledge of the vegetation types and the spatial variability of AGB in the study area [71].

Developing an AGB estimation model and applying it for mapping AGB in large areas involves several uncertainties. One of the main problems is that SAR imagery underestimates AGB in areas of high forest biomass, due to saturation of backscatter in dense vegetation [29]. Our results showed a slight under estimation for high values of biomass (Fig. 7, bias 0 − 1.04), even though we used texture measures and climatic data to improve the AGB estimation (Fig. 3a). However, compared to the studies of Rodriguez-Veiga et al. [8] and Cartus el al. [10], the underestimation at high levels of biomass was considerably lower in this study (Fig. 8b, Fig/bias = − 50.3 and − 40.4 respectively). The ranking of variables in random forest indicated that CWD and some texture measures contributed more than HH polarization to explain the variation in AGB estimation (Additional file 9: Fig. S6). These results suggest that the saturation in the SAR imagery can be reduced by using relevant environmental data to predict biomass –in this case, water deficit, which is one of the most important factors limiting forest growth in tropical dry forests [19, 20]. Additionally, the lower underestimation error of AGB at high biomass values in this study may be partly attributed to the use of texture measurements, which can capture variation in horizontal forest structure attributes of paramount importance for biomass estimation, such as differences in tree height and crown diameter of patches of forest with different stand age [37–39]. Therefore, the use of these two groups of variables, together with backscatter may have improved the biomass estimation. In contrast, the results of this study showed an overestimation of AGB at smaller biomass values, as commonly reported by several studies [6, 38]. This overestimation may be caused by open areas or non-forest areas (with almost null AGB values) contributing to the backscatter signal. One way to further improve AGB estimation and reduce the overestimation of AGB at small levels of biomass is by adding time series of remotely sensed data to capture forest changes in reflectance values, or estimating forest stand age, especially early in succession [26].

When using random forest to estimate AGB it is important to test for spatial autocorrelation in the residuals of the models, since deviations from the assumption of independence (i.e. no autocorrelation) can result in declaring significant effects when there are none [72]. Here, we found no significant spatial autocorrelation in all random forest models tested. However, there are some statistical analysis that combine regression models with ordinary kriging of regression residuals, to take into consideration both the autocorrelation and the associations between AGB and explanatory variables [73]. In a similar way, random forest has been combined with model residuals for mapping the spatial distribution of AGB [74].

To put our results in perspective, we compared the performance of the AGB map of this study with those from two previous studies [8, 10]. Although, the three maps were generated using the same source of field data (the national forest inventory) and similar remotely sensed imagery, the map validation analysis indicated that our map performed better. Our map had higher model fit values and lower relative RMSE (R^2^ = 0.44 and  %RMSE = 32.3) compared to the previous studies of Cartus (R^2^ = 0.32 and  %RMSE = 69.7) and Rodriguez-Veiga (R^2^ = 0.17 and  %RMSE = 69.9). Besides, the ranges and mean AGB values differed among the three maps. The map of this study displayed significantly higher values of AGB in comparison to the previous studies and showed more similar ranges and mean AGB values compared to the reference data (Fig. 8a). These differences are mainly due to the processes of correcting NFI plot data, for not considering small trees and for temporal differences between imagery data acquisition and field measurements. On the other hand, the three maps underestimate AGB for biomass values larger than 100 Mg ha^−1^ and overestimate at low levels of biomass. However, the underestimation of AGB was lower in this study compared to previous ones (Fig. 8b, see bias in Fig. 7). These differences could be explained by the fact that we generated models for the three main types of TDF in the Yucatán peninsula using local wood density values and allometric equations. We also used relevant environmental variables to predict biomass, such as CWD, one of the most important factors limiting growth in tropical dry forests [19, 20]. In addition, we used texture measures to predict biomass, which are important for capturing variation in horizontal forest structure among patches of different successional age [28]. Finally, the three studies reported higher error for the deciduous than for the semi-deciduous and semi-evergreen forests. This may be due to the differences in sample size between those forests. Although we tested an approach to improve the accuracy of predictions of AGB by using LiDAR plots (in a two-stage upscaling method), we did not succeed because LiDAR plots could not capture the range of AGB variability in these forests.

## Conclusion

We present a potentially useful approach for mapping AGB in tropical forests using random forest models with AGB estimated from field plots. By addressing the main sources of errors encountered when mapping AGB and applying ecological knowledge to plot data we improved the accuracy of AGB maps for the tropical dry forests of the Yucatan peninsula. Small stems (< 7.5 cm DBH) make a significant contribution to AGB in TDF, additionally, since the NFI plots were measured over a 6-year time window, annual increases in AGB mean that the temporal difference between field and remotely sensed measurements should be accounted for. By correcting plot data for small stems and the temporal difference between field and remotely sensed measurements, we reduced relative error of biomass estimates by 12.2%.

In order to minimize the error associated with small sample size, we increased the sample size by combining both field and LiDAR plots or using LiDAR plots to train a model for predicting AGB in larger areas. However, we found a better performance of the approach that used only field plots (R^2^ = 0.44 and  %RMSE = 32.1), compared to the approach that combined both data sets (R^2^ = 0.35 and  %RMSE = 41.8) because the LiDAR plots showed a poor performance (R^2^ = 0.26 and  %RMSE = 63.4). This low performance contrasted with the good performance of the model that related field AGB measurements and LiDAR metrics, indicating that LiDAR plots, which covered less than 1% of the study area, did not capture the range of AGB variability of our study forests.

Our results also showed that the inclusion of climatic data and texture measures from ALOS PALSAR reduced the saturation effect, since a relevant environmental variable, such as CWD is also highly related to forest biomass. Similarly, texture measures can capture variations in forest structure over the study area that are related to biomass. Finally, our results suggest that, understanding the main sources of errors during the process of estimating AGB, as well as using of some approaches to correct those errors, improved the accuracy of AGB estimates compared to previous studies.

## Supplementary information

**Additional file 1: Table S1.** List of wood density values of plant species and the corresponding references.

**Additional file 2: Table S2.** Factors to correct National Forest Inventory plots for failing to consider small (DBH < 7.5 cm) trees in each percentile class and forest type.

**Additional file 3: Fig S1.** Above ground biomass as a function of successional stand age for the tropical dry forests of our study area: deciduous and semi-deciduous (a) and semi-evergreen (b).

**Additional file 4: Fig. S2.** Climatic water deficit map of the Yucatan peninsula calculated with interpolated evapotranspiration and rainfall monthly maps (a). Area for interpolating climatic variables (rainfall, temperature and evapotranspiration) from 425 meteorological stations.

**Additional file 5: Table S3.** Regression parameters of the best model used to estimate aboveground biomass from LiDAR data.

**Additional file 6: Fig S3.** Results of cross validation analyses of the regression model between AGB and LiDAR data. The red line shows 1:1 reference line and the dashed line show the regression line.

**Additional file 7: Fig S4.** Frequency histograms and maps of estimated AGB in a 3600 km^2^ window of tropical dry semi-deciduous forest in this study (a, d), in the study of Cartus et al. [9] (b, e) and in the study of Rodriguez-Veiga et al. [7] (c, f).

**Additional file 8: Fig S5.** Frequency histograms of AGB from field plots for three types of tropical dry forests: deciduous (a), semi-deciduous (b) and semi-evergreen (c); and of AGB estimated from LiDAR plots: deciduous (d), semi-deciduous (e) and semi-evergreen forests (f).

**Additional file 9: Fig S6.** Importance of random forest predictors for modelling AGB from backscatter HH and HV polarization, normalized difference backscatter index (NDBI) and texture measures from ALOS PALSAR as well as climatic water deficit (CWD).

## Data Availability

The ALOS PALSAR data used in this study was downloaded from (https://www.eorc.jaxa.jp/ALOS/en/top/obs_top.htm). The LiDAR data can be accessed at (https://gliht.gsfc.nasa.gov/). Data from national forest inventory in Mexico can be obtained by request to CONAFOR (Comisión Nacional Forestal, https://www.gob.mx/conafor). The additional datasets used in this manuscript are available upon request to corresponding author.

## Abbreviations

AGB: Aboveground biomass
TDF: Tropical dry forests
DBH: Diameter at breast height
NFI: National forest inventory
IMC: Intensive monitoring carbon
NDBI: Normalized difference backscatter index
CWD: Climatic water deficit
ALOS–PALSAR: Advanced land observing satellite–phased array type l-band synthetic aperture radar
LiDAR: Light detection and ranging
SAR: Synthetic aperture radar
SRTM: Shuttle radar topography mission
RMSE: Root mean square error
%RMSE: Relative root mean square error
R^2^: Coefficient of determination
