# Supplementary material for: Improving aboveground biomass maps of tropical dry forests by integrating LiDAR, ALOS PALSAR, climate and field data
Source: Carbon Balance Manag. 2020 Jul 29;15:15. doi: 10.1186/s13021-020-00151-6 (PMC7392681; doi:10.1186/s13021-020-00151-6)
Supplement: Supplementary file 1 — Additional file 1: Table S1. List of wood density values of plant species and the corresponding references. [file 13021_2020_151_MOESM1_ESM.docx]

Table S1. List of wood density values of plant species and the corresponding references.

| **Species** | **Density (g/cm3)** | **Reference** |
| --- | --- | --- |
| *Acacia angustissima* | 0.85 | Chave et al. [1] |
| *Acacia cochliacantha* | 0.94 | Barajas Morales [2] |
| *Acacia gaumeri* | 0.9 | Sanaphre Villanueva et al. [3] |
| *Acacia glomerosa* | 0.47 | Erdoisa Sordo and Castillo Morales [4] |
| *Acacia hindsii* | 0.78 | Barajas Morales [2] |
| *Acacia pennatula* | 1.06 | Sanaphre Villanueva et al. [3] |
| *Adelia oaxacana* | 0.87 | Barajas Morales [2] |
| *Albizia tomentosa* | 0.45 | Hidayat and Simpson [5] |
| *Alseis yucatanensis* | 0.6 | Chave et al. [1] |
| *Alvaradoa amorphoides* | 0.54 | Reyes-Garcia et al. [6] |
| *Amphilophium paniculatum var. molle* | 0.58 | Sanaphre Villanueva et al. [3] |
| *Amphilophium paniculatum var. Paniculatum* | 0.58 | Sanaphre Villanueva et al. [3] |
| *Apoplanesia paniculata* | 0.7 | Reyes-Garcia et al. [6] |
| *Astronium graveolens* | 0.87 | Silva Guzman et al. [7] |
| *Bauhinia divaricata* | 0.75 | Sanaphre Villanueva et al. [3] |
| *Bauhinia ungulata* | 0.94 | Sanaphre Villanueva et al. [3] |
| *Bourreria pulchra* | 0.78 | Sanaphre Villanueva et al. [3] |
| *Brosimum alicastrum* | 0.75 | Silva Guzman et al. [7] |
| *Bucida buceras* | 0.93 | FAO [8] |
| *Bunchosia swartziana* | 0.58 | Sanaphre Villanueva et al. [3] |
| *Bursera instabilis* | 0.24 | Barajas Morales [2] |
| *Bursera simaruba* | 0.36 | Sanaphre Villanueva et al. [3] |
| *Byrsonima crassifolia* | 0.63 | Tamarit Urias [9] |
| *Byrsonima spp.* | 0.64 | FAO [8] |
| *Caesalpinia gaumeri* | 0.9 | Sanaphre Villanueva et al. [3] |
| *Caesalpinia platyloba* | 0.94 | Tamarit Urias [9] |
| *Caesalpinia pulcherrima* | 0.84 | Barajas Morales [2] |
| *Caesalpinia spp.* | 1.05 | FAO [8] |
| *Calyptranthes pallens* | 0.86 | Sanaphre Villanueva et al. [3] |
| *Capparis verrucosa* | 0.86 | Barajas Morales [2] |
| *Casearia corymbosa* | 0.67 | Barajas Morales [2] |
| *Casearia emarginata* | 0.62 | Reyes et al. [10] |
| *Cecropia obtusifolia* | 0.31 | Erdoisa Sordo and Castillo Morales [4] |
| *Cecropia peltata* | 0.3 | FAO [8] |
| *Cedrela odorata* | 0.45 | FAO [8] |
| *Ceiba aesculifolia* | 0.48 | Barajas Morales [2] |
| *Ceiba pentandra* | 0.27 | Silva Guzman et al. [7] |
| *Ceiba schotti* | 0.48 | Reyes-Garcia et al. [6] |
| *Cesalpinea gaumeri* | 0.74 | Reyes-Garcia et al. [6] |
| *Chloroleucon mangense* | 0.59 | Sanaphre Villanueva et al. [3] |
| *Chlorophora tinctoria* | 0.96 | Barajas Morales [2] |
| *Cnidoscolus aconitifolius* | 0.29 | Sanaphre Villanueva et al. [3] |
| *Coccoloba acapulcensis* | 0.65 | Sanaphre Villanueva et al. [3] |
| *Coccoloba barbadensis* | 0.71 | Barajas Morales [2] |
| *Coccoloba diversifolia* | 0.8 | Chave et al. [1] |
| *Coccoloba spicata* | 0.86 | Sanaphre Villanueva et al. [3] |
| *Cochlospermum vitifolium* | 0.23 | Sanaphre Villanueva et al. [3] |
| *Cojoba arborea* | 0.8 | Silva Guzman et al. [7] |
| *Colubrina elliptica* | 0.8 | Chave et al. [1] |
| *Cordia alliodora* | 0.53 | Silva Guzman et al. [7] |
| *Cordia dodecandra* | 0.96 | Silva Guzman et al. [7] |
| *Cordia gerascanthus* | 0.75 | Chave et al. [1] |
| *Croton glabellus* | 1 | Tamarit Urias [9] |
| *Croton nitens* | 0.53 | Barajas Morales [2] |
| *Croton reflexifolius* | 1.09 | Sanaphre Villanueva et al. [3] |
| *Cydista diversifolia* | 0.51 | Sanaphre Villanueva et al. [3] |
| *Cydista potosina* | 0.51 | Sanaphre Villanueva et al. [3] |
| *Dalbergia glabra* | 0.47 | Sanaphre Villanueva et al. [3] |
| *Dendropanax arboreus* | 0.53 | Silva Guzman et al. [7] |
| *Diospyros anisandra* | 0.94 | Sanaphre Villanueva et al. [3] |
| *Diospyros cuneata* | 0.82 | Sanaphre Villanueva et al. [3] |
| *Diospyros tetrasperma* | 0.82 | Sanaphre Villanueva et al. [3] |
| *Diospyros yucatanensis ssp spectabilis* | 0.78 | Sanaphre Villanueva et al. [3] |
| *Diospyros yucatanensis ssp yucatanensis* | 0.9 | Sanaphre Villanueva et al. [3] |
| *Drypetes lateriflora* | 0.72 | Fuentes Salinas et al. [18] |
| *Ebenopsis ebano* | 1.06 | Zizumbo Cortes [11] |
| *Enterolobium cyclocarpum* | 0.45 | Silva Guzman et al. [7] |
| *Erythrina sp.* | 0.23 | FAO [8] |
| *Erythroxylum rotundifolium* | 0.9 | Sanaphre Villanueva et al. [3] |
| *Eugenia axillaris* | 0.77 | Sanaphre Villanueva et al. [3] |
| *Eugenia buxifolia* | 0.91 | Sanaphre Villanueva et al. [3] |
| *Exostema caribaeum* | 0.88 | Sanaphre Villanueva et al. [3] |
| *Ficus cotinifolia* | 0.4 | Barajas Morales [2] |
| *Ficus sp.* | 0.32 | FAO [8] |
| *Gliricidia sepium* | 0.64 | Barajas Morales [2] |
| *Guazuma ulmifolia* | 0.5 | FAO [8] |
| *Guettarda elliptica* | 0.83 | Sanaphre Villanueva et al. [3] |
| *Guettarda gaumeri* | 0.74 | Yam Uicab, O. [12] |
| *Gymnanthes lucida* | 1.1 | Chave et al. [1] |
| *Gymnopodium floribundum* | 0.79 | Sanaphre Villanueva et al. [3] |
| *Gyrocarpus americanus* | 0.32 | Hidayat and Simpson [5] |
| *Haematoxylon brasiletto* | 1.1 | Jimenez et al. [13] |
| *Havardia albicans* | 0.74 | Yam Uicab, O. [12] |
| *Heliocarpus donnell-smithii* | 0.16 | Chave et al. [1] |
| *Heteropterys brachiata* | 0.542 | Sanaphre Villanueva et al. [3] |
| *Hippocratea excelsa* | 0.74 | Sanaphre Villanueva et al. [3] |
| *Inga vera* | 0.59 | FAO [8] |
| *Ipomoea wolcottiana* | 0.57 | Barajas Morales [2] |
| *Jacaratia mexicana* | 0.16 | Barajas Morales [2] |
| *Jacquinia macrocarpa* | 0.61 | Reyes-Garcia et al. [6] |
| *Jatropha gaumeri* | 0.43 | Sanaphre Villanueva et al. [3] |
| *Karwinskia humboldtiana* | 0.86 | Sanaphre Villanueva et al. [3] |
| *Krugiodendron ferreum* | 0.91 | Correa Mendez [14] |
| *Lachmellea speciosa* | 0.73 | FAO [8] |
| *Laetia thamnia* | 0.66 | Chave et al. [1] |
| *Leucaena lanceolata* | 0.94 | Barajas Morales [2] |
| *Leucaena leucocephala* | 0.76 | Sanaphre Villanueva et al. [3] |
| *Lonchocarpus castilloi* | 0.83 | Silva Guzman et al. [7] |
| *Lonchocarpus hondurensis* | 0.83 | Chave et al. [1] |
| *Lonchocarpus parviflorus* | 0.89 | Barajas Morales [2] |
| *Lonchocarpus rugosus* | 0.95 | Sanaphre Villanueva et al. [3] |
| *Lonchocarpus sp.* | 0.69 | Reyes et al. [10] |
| *Lonchocarpus xuul* | 0.82 | Sanaphre Villanueva et al. [3] |
| *Luehea candida* | 0.91 | Barajas Morales [2] |
| *Luehea speciosa* | 0.72 | Sanaphre Villanueva et al. [3] |
| *Lysiloma divaricatum* | 0.73 | Correa Mendez [14] |
| *Lysiloma latisiliquum* | 0.55 | Sanaphre Villanueva et al. [3] |
| *Machaonia lindeniana* | 0.99 | Sanaphre Villanueva et al. [3] |
| *Maclura tinctoria* | 0.71 | Echenique-Manrique [15] |
| *Malpighia glabra* | 0.91 | Sanaphre Villanueva et al. [3] |
| *Manilkara zapota* | 0.88 | Hidayat and Simpson [5] |
| *Mansoa verrucifera* | 0.5 | Sanaphre Villanueva et al. [3] |
| *Melicoccus oliviformis* | 0.71 | Sanaphre Villanueva et al. [3] |
| *Metopium brownei* | 0.8 | Tamarit Urias [9] |
| *Mimosa bahamensis* | 0.91 | Sanaphre Villanueva et al. [3] |
| *Nectandra coriacea* | 0.51 | FAO [8] |
| *Nectandra salicifolia* | 0.46 | Barajas Morales [2] |
| *Neea psychotrioides* | 0.71 | Sanaphre Villanueva et al. [3] |
| *Neomillspaughia emarginata* | 0.8 | Sanaphre Villanueva et al. [3] |
| *Pachira aquatica* | 0.5 | Torelli [16] |
| *Parathesis cubana* | 0.62 | Chave et al. [1] |
| *Phyllostylon brasilense* | 0.72 | Reyes-Garcia et al. [6] |
| *Pimenta dioica* | 0.96 | Barajas Morales [2] |
| *Piscidia piscipula* | 0.78 | Sanaphre Villanueva et al. [3] |
| *Pisonia aculeata* | 0.43 | Sanaphre Villanueva et al. [3] |
| *Pithecellobium dulce* | 0.72 | Reyes-Garcia et al. [6] |
| *Platymiscium spp.* | 0.71 | FAO [8] |
| *Platymiscium yucatanum* | 0.9 | Sanaphre Villanueva et al. [3] |
| *Pouteria aff. Campechiana* | 0.75 | Sotomayor Castellanos et al. [17] |
| *Pouteria sapota* | 0.81 | Barajas Morales [2] |
| *Protium copal* | 0.59 | Sotomayor Castellanos et al. [17] |
| *Pseudobombax ellipticum* | 0.44 | Torelli [16] |
| *Psidium sartorianum* | 0.85 | Sanaphre Villanueva et al. [3] |
| *Pterocarpus rohrii* | 0.41 | FAO [8] |
| *Randia longiloba* | 0.74 | Yam Uicab, O. [12] |
| *Randia obcordata* | 0.7 | Reyes et al. [10] |
| *Samyda yucatanensis* | 0.72 | Sanaphre Villanueva et al. [3] |
| *Sapindus saponaria* | 0.66 | Correa Mendez [14] |
| *Sapium lateriflorum* | 0.5 | Tamarit Urias [9] |
| *Sapium laurocerasus* | 0.38 | FAO [8] |
| *Semialarium mexicanum* | 0.74 | Sanaphre Villanueva et al. [3] |
| *Senna atomaria* | 0.7 | Sanaphre Villanueva et al. [3] |
| *Senna racemosa* | 0.68 | Sanaphre Villanueva et al. [3] |
| *Sideroxylon sp.* | 0.62 | Reyes-Garcia et al. [6] |
| *Sideroxylon obtusifolium* | 1.07 | Sanaphre Villanueva et al. [3] |
| *Simarouba amara* | 0.43 | Silva Guzman et al. [7] |
| *Simarouba glauca* | 0.46 | Torelli [16] |
| *Spondias mombin* | 0.4 | Silva Guzman et al. [7] |
| *Spondias purpurea* | 0.31 | Barajas Morales [2] |
| *Spondias radlkoferi* | 0.56 | Barajas Morales [2] |
| *Swartzia cubensis* | 1.12 | Silva Guzman et al. [7] |
| *Swartzia guatemalensis* | 0.89 | Barajas Morales [2] |
| *Swietenia macrophylla* | 0.42 | Torelli [16] |
| *Tabebuia chrysantha* | 0.75 | Sanaphre Villanueva et al. [3] |
| *Tabebuia guayacan* | 0.82 | FAO [8] |
| *Tabebuia rosea* | 0.6 | Silva Guzman et al. [7] |
| *Tabernaemontana alba* | 0.45 | Sanaphre Villanueva et al. [3] |
| *Talisia floressi* | 0.84 | Reyes et al. [10] |
| *Talisia olivaeformis* | 0.71 | Sanaphre Villanueva et al. [3] |
| *Thouinia paucidentata* | 0.94 | Sanaphre Villanueva et al. [3] |
| *Trema micrantha* | 0.35 | Chave et al. [1] |
| *Trichilia glabra* | 0.74 | Yam Uicab, O. [12] |
| *Trichilia hirta* | 0.55 | Chave et al. [1] |
| *Trichilia martiana* | 0.47 | Barajas Morales [2] |
| *Trichilia moschata* | 0.88 | Barajas Morales [2] |
| *Trichilia pallida* | 0.69 | Barajas Morales [2] |
| *Trichilia propingua* | 0.58 | FAO [8] |
| *Trophis racemosa* | 0.78 | Barajas Morales [2] |
| *Vitex gaumeri* | 0.65 | Sanaphre Villanueva et al. [3] |
| *Ximenia americana* | 0.66 | Sanaphre Villanueva et al. [3] |
| *Zanthoxylum caribaeum* | 0.97 | Sanaphre Villanueva et al. [3] |
| *Zanthoxylum fagara* | 0.65 | Fuentes Salinas et al. [18] |
| *Zapoteca formosa* | 0.67 | Sanaphre Villanueva et al. [3] |
| *Zuelania guidonia* | 0.61 | Torelli [16] |

REFERENCES

1. Chave J, Condit R, Lao S, Caspersen JP, Foster RB, and Hubbell SP. Spatial and temporal variation of biomass in a tropical forest: results from a large census plot in Panama. Journal of Ecology. 2003; 91: 240-252.
2. Barajas-Morales J. Wood specifc gravity in species from two tropical forests in Mexico. IAWA Bulletin n.s. 1987; 8(2): 143 – 148.
3. Sanaphre-Villanueva L, Dupuy JM, Andrade JL, Reyes-Garcia C, Pax H. Functional diversity of small and large trees along secondary succession in a tropical dry forest. Forests. 2016; 7, 163.
4. Edroisa Sordo JJ, Castillo-Morales MI. Susceptibilidad de impregnacion con preservadores de cincuenta especies maderables mexicanas. La madera y sus usos 22. Boletin tecnico. LACITEMA. 1992; 17.
5. Hidayat S, Simpson WT. Use of green moisture content and basic specific gravity to group tropical woods for Kiln Drying. Res. Note. US Department of Agriculture, Forest Service, Forest Products Laboratory. 1994; 0-39.
6. Reyes-Garcia C, Andrade JL, Sima JL, Us-Santamaria R, Jackson P. Sapwood to heartwood ratio affects whole-tree water use in dry forest legume and non-legume trees. Trees. 2012; 26 1317-1330.
7. Silva Guzman JA, Fuentes-Talavera FJ, Rodriguez-Anda R, Torres-Andrade PA, Lomelí-Ramirez MA, Ramos-Quirarte J, Waitkus C, Richter HG. Fichas de propiedades tecnológicas y usos de maderas nativas se México e importadas. Departamento de Madera, Celulosa y Papel, Universidad de Guadalajara y Comisión Nacional Forestal. 2010; 208.
8. FAO (Food and Aagriculture Organization). Estimating Biomass and Biomass Change of Tropical Forests: A Primer. FAO Forestry Paper – 134. 1997.
9. Tamarit-Urias JC. Determinación de los índices de calidad de pulpa para papel de 132 maderas latifoliadas. Madera y Bosques 1995; 2(2):29-41
10. Reyes G, Brown S, Chapman J, Lugo A. Wood densities of tropical Tree species. Gen. Tech. Rep. SO–88. United States Department of Agriculture Forest Service. Forest Experiment Station. New Orleans, LA, USA. 1992; 15 p.
11. Zizumbo-Cortes F. Estudio Tecnologico de Pithecellobium ebano (Benth.) Berlan., como fundamento para su aprovechamiento racional. Tesis de Maestria. Facultad de Ingenieria en Tecnologia de la Madera. Universidad Michoacana de San Nicolas de Hidalgo. 1998; 159.
12. Yam Uicab, O. Estimación de la distribución espacial del carbono y la biomasa vegetal area en un paisaje de selva mediana del sur de Yucatán. Tesis de Maestria. Posgrado en Ciencias Biológicas, Centro de Investigación Cientifica de Yucatan A.C. 2012. 80.
13. Jimenez-Ferrer G, Lopez-Carmona M, Nahed-Toral J, Ochoa-Gaona S, Jong B. Arboles y arbustos forrajeros de la region norte-tzotzil de Chiapas, Mexico. Vet Mex. 2008; 39(2):199-2213.
14. Correa-Mendez, F. Factibilidad tecnologica de aprovechamiento para tableros aglomerados de 16 especies de maderas del Edo. de Tamaulipas, México. Tesis de Maestria. Universidad Autonoma de Chapingo. 2006.
15. Echenique-Manrique R. Descripcion, características y usos de 25 maderas tropicales mexicanas. Maderas de Mexico. 1970; 237.
16. Torelli N. Promotional study of 43 Mexican tropical timber species. Secretaria de Agricultura y Recursos Hidraulicos. 1982.
17. Sotomayor-Castellanos JR. Tabla FICTEMA de clasificacion de caracteristicas mecanicas de maderas mexicanas. FITECMA. UMSNH. 2008.
18. Fuentes-Salinas M, Correa-Mendez A, Borja-de la Rosa A, Corona-Ambriz A. Caracteristicas tecnologicas de 16 maderas del estado de Tamaulipas que influyen en la fabricacion de tableros de particulas y de fibras. Revista Chapingo Serie Ciencias Forestales y del Ambiente. 2008; 14(1): 65-71.
