# Supplementary material for: Improving aboveground biomass maps of tropical dry forests by integrating LiDAR, ALOS PALSAR, climate and field data
Source: Carbon Balance Manag. 2020 Jul 29;15:15. doi: 10.1186/s13021-020-00151-6 (PMC7392681; doi:10.1186/s13021-020-00151-6)
Supplement: Supplementary file 2 — Additional file 2: Table S2. Factors to correct National Forest Inventory plots for failing to consider small (DBH < 7.5 cm) trees in each percentile class and forest type. [file 13021_2020_151_MOESM2_ESM.docx]

Table S2. Factors to correct National Forest Inventory plots for failing to consider small (DBH < 7.5 cm) trees in each percentile class and forest type.

| **Percentile** | **Type of forest** | **AGB (Mg/ha)** | | **Correction factor** |
| --- | --- | --- | --- | --- |
|  |  | **Range** | |  |
| [0 - 10] | Deciduos | < | 37.5 | 1.56 |
|  | Semi-deciduos | < | 17.8 | 1.87 |
|  | Semi-evergreen | < | 66.9 | 1.93 |
| (10 - 25] | Deciduos | 37.6 | 45.7 | 1.45 |
|  | Semi-deciduos | 17.8 | 77.8 | 1.55 |
|  | Semi-evergreen | 67 | 185.3 | 1.29 |
| (25 - 50] | Deciduos | 45.8 | 69.4 | 1.25 |
|  | Semi-deciduos | 77.9 | 110.3 | 1.36 |
|  | Semi-evergreen | 185.4 | 212.0 | 1.15 |
| (50 - 75] | Deciduos | 69.5 | 79.7 | 1.22 |
|  | Semi-deciduos | 110.4 | 142.2 | 1.19 |
|  | Semi-evergreen | 212.1 | 254.3 | 1.14 |
| (75 - 100] | Deciduos | > | 79.8 | 1.17 |
|  | Semi-deciduos | > | 142.3 | 1.11 |
|  | Semi-evergreen | > | 254.4 | 1.09 |
