# Supplementary figures and images for: Improving aboveground biomass maps of tropical dry forests by integrating LiDAR, ALOS PALSAR, climate and field data
Source: Carbon Balance Manag. 2020 Jul 29;15:15. doi: 10.1186/s13021-020-00151-6 (PMC7392681; doi:10.1186/s13021-020-00151-6)

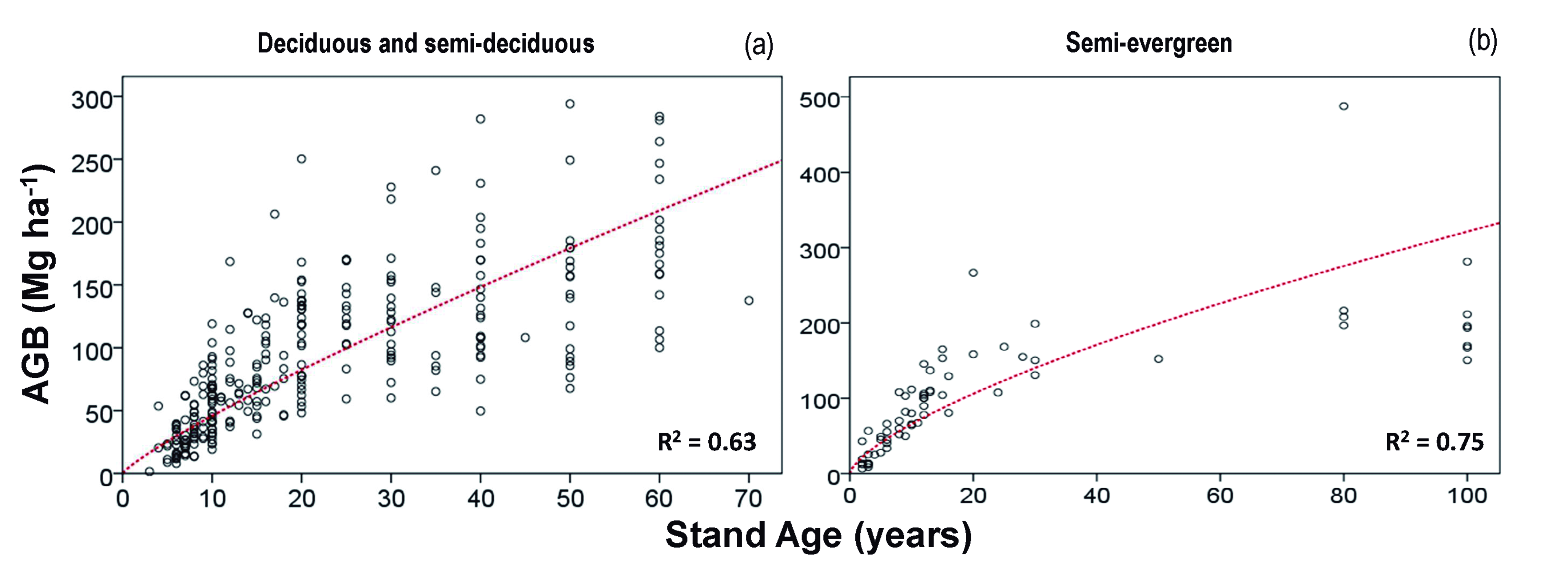

Supplement: Supplementary file 3 — Additional file 3: Fig S1. Above ground biomass as a function of successional stand age for the tropical dry forests of our study area: deciduous and semi-deciduous (a) and semi-evergreen (b). [file 13021_2020_151_MOESM3_ESM.tif]

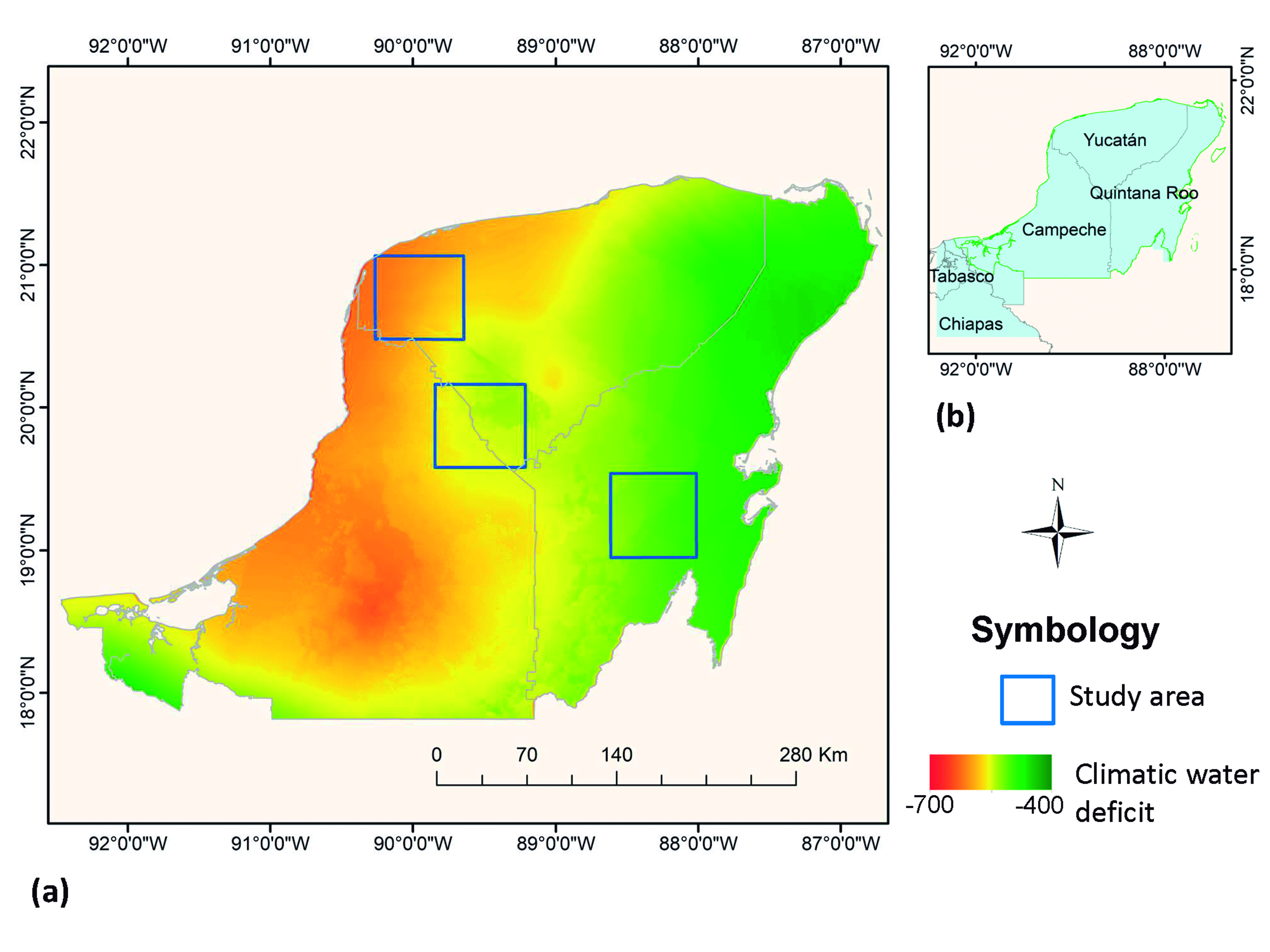

Supplement: Supplementary file 4 — Additional file 4: Fig. S2. Climatic water deficit map of the Yucatan peninsula calculated with interpolated evapotranspiration and rainfall monthly maps (a). Area for interpolating climatic variables (rainfall, temperature and evapotranspiration) from 425 meteorological stations. [file 13021_2020_151_MOESM4_ESM.tiff]

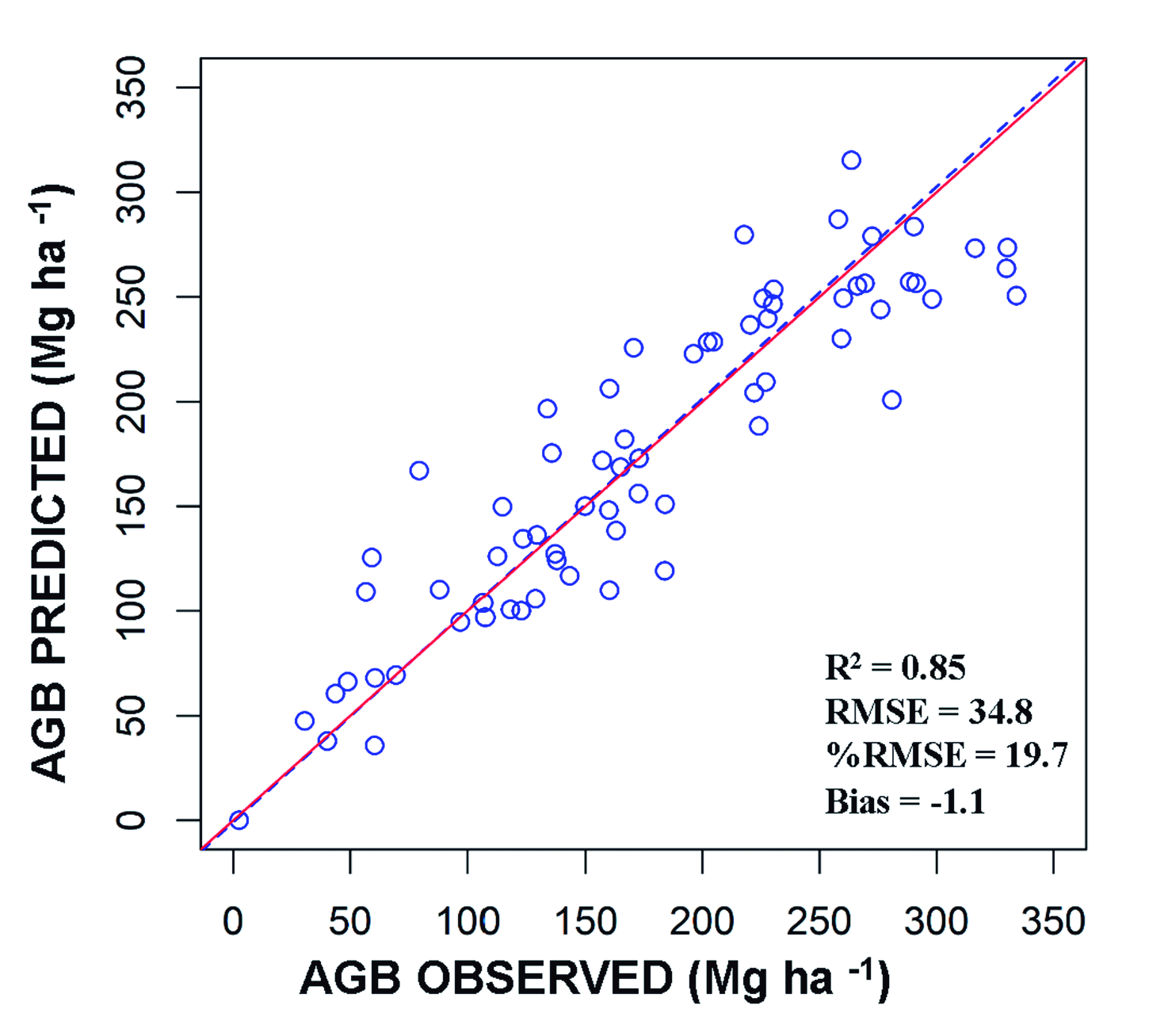

Supplement: Supplementary file 6 — Additional file 6: Fig S3. Results of cross validation analyses of the regression model between AGB and LiDAR data. The red line shows 1:1 reference line and the dashed line show the regression line. [file 13021_2020_151_MOESM6_ESM.tif]

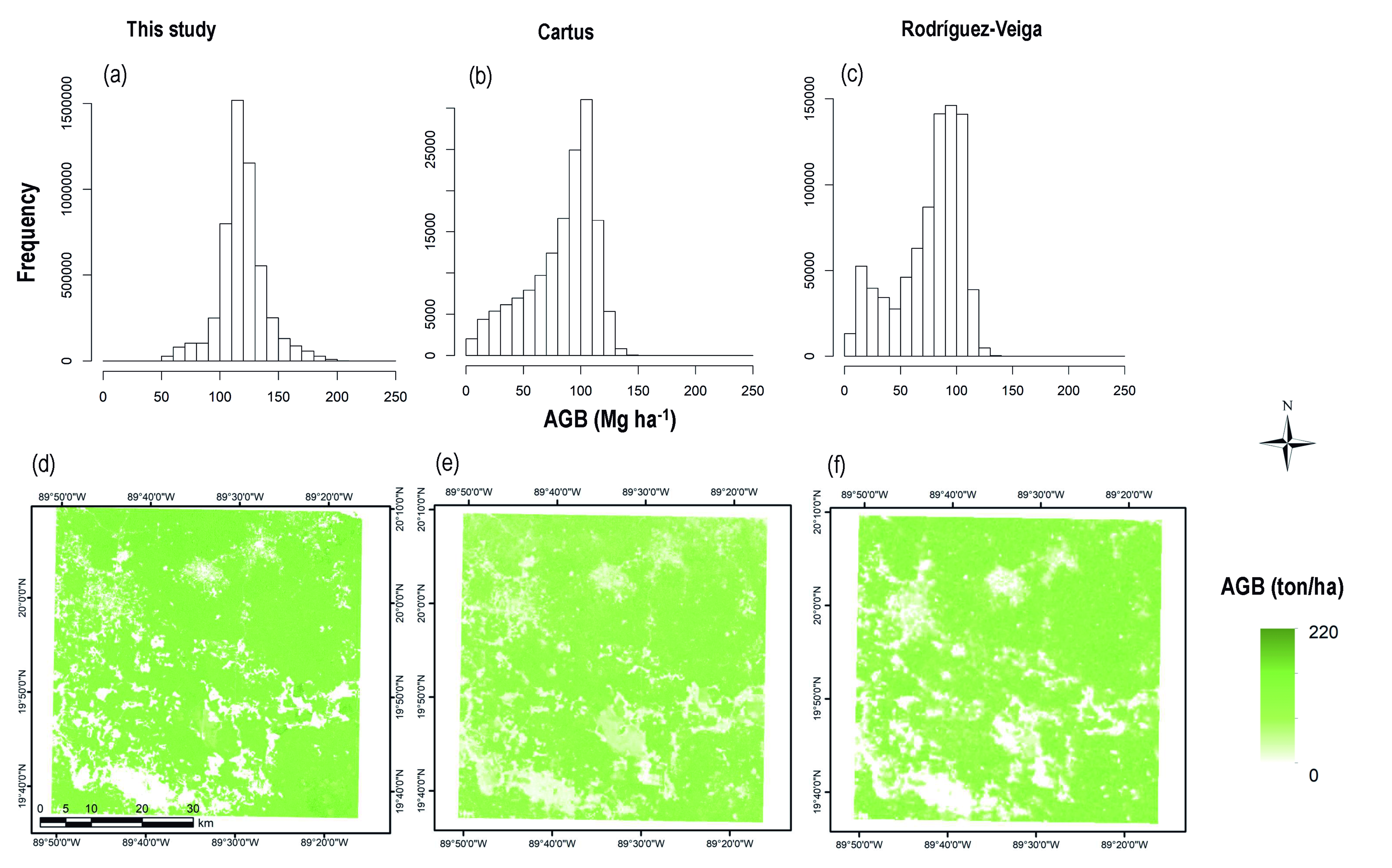

Supplement: Supplementary file 7 — Additional file 7: Fig S4. Frequency histograms and maps of estimated AGB in a 3600 km2 window of tropical dry semi-deciduous forest in this study (a, d), in the study of Cartus et al. [9] (b, e) and in the study of Rodriguez-Veiga et al. [7] (c, f). [file 13021_2020_151_MOESM7_ESM.tif]

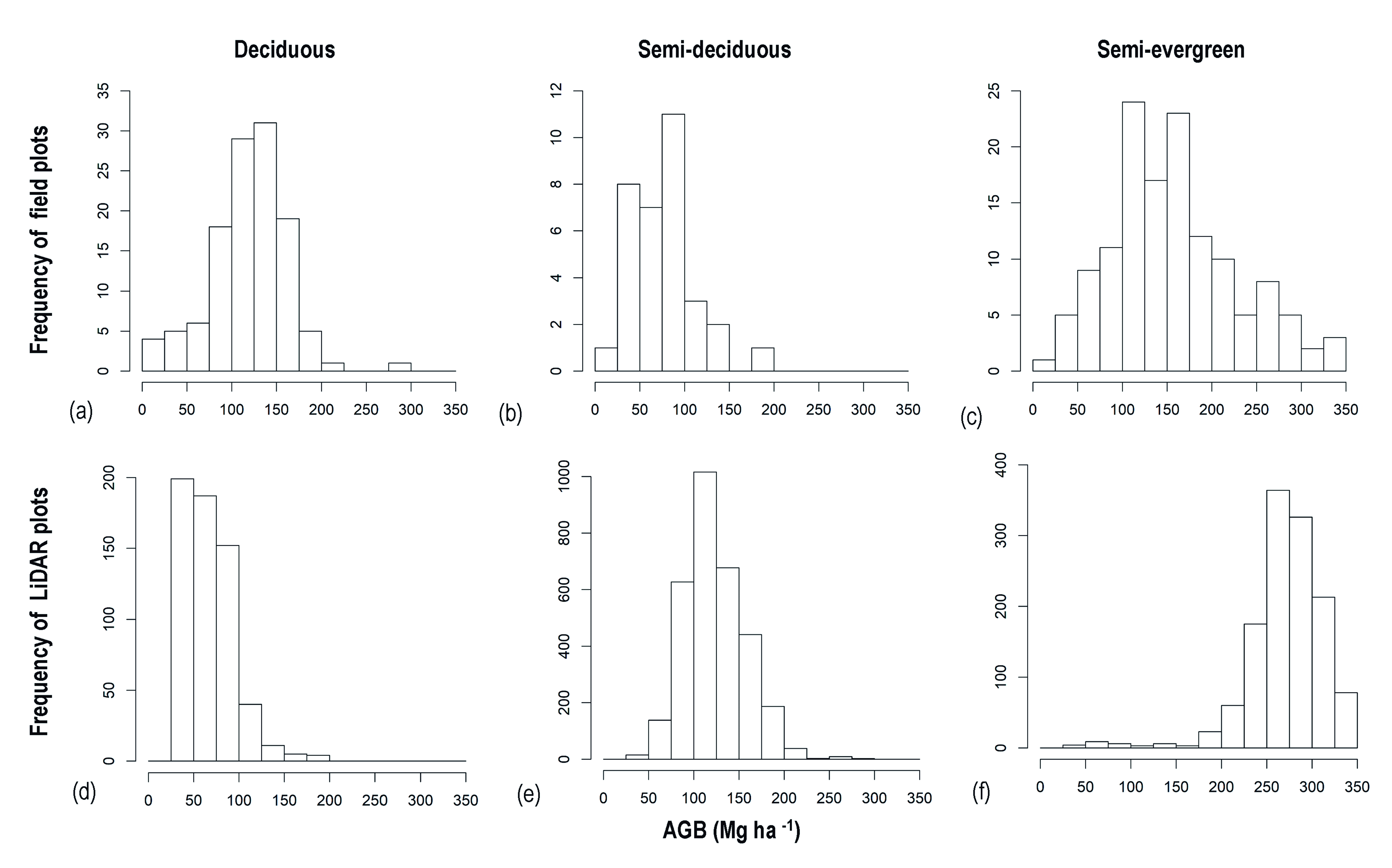

Supplement: Supplementary file 8 — Additional file 8: Fig S5. Frequency histograms of AGB from field plots for three types of tropical dry forests: deciduous (a), semi-deciduous (b) and semi-evergreen (c); and of AGB estimated from LiDAR plots: deciduous (d), semi-deciduous (e) and semi-evergreen forests (f). [file 13021_2020_151_MOESM8_ESM.tif]

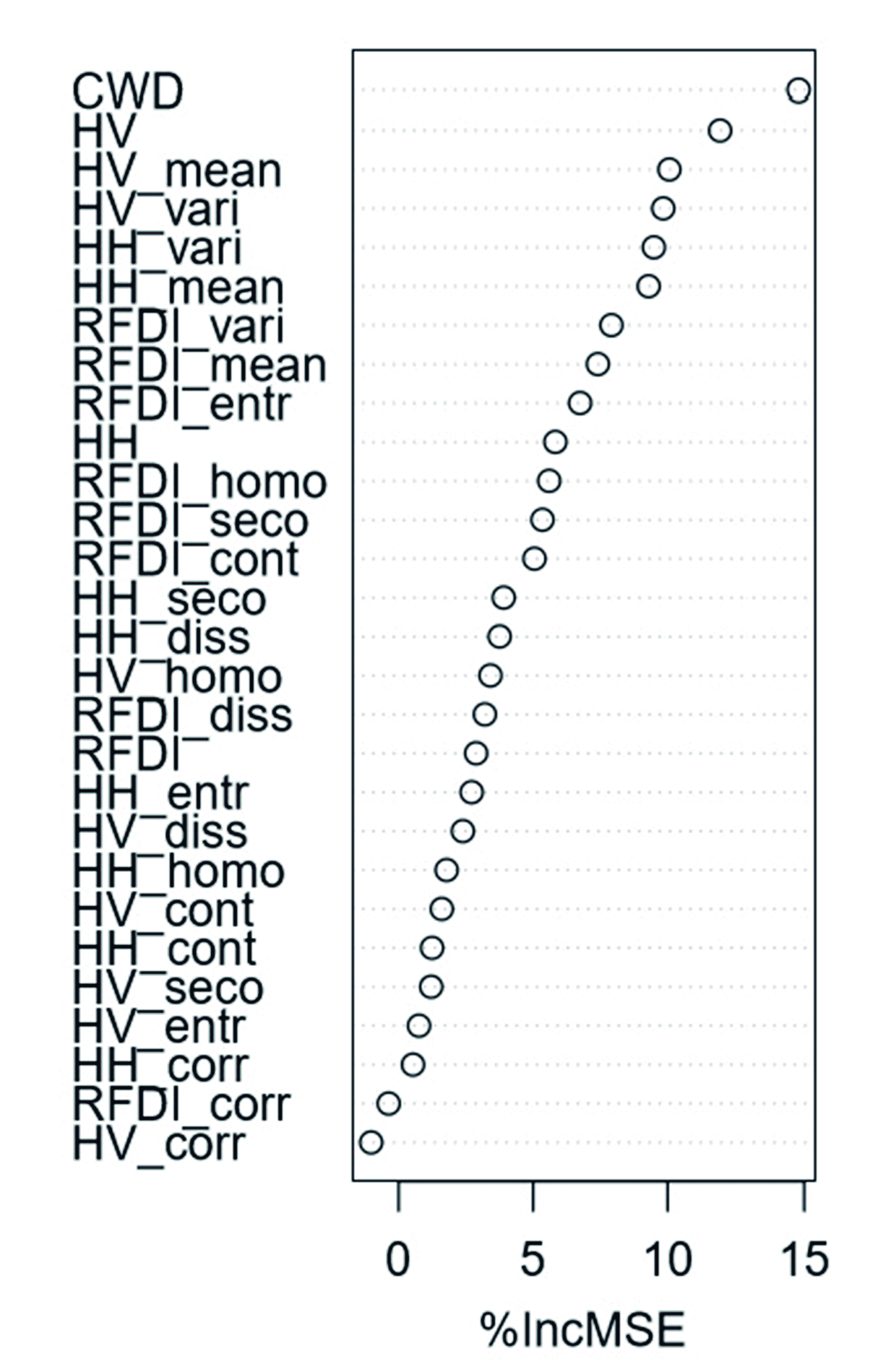

Supplement: Supplementary file 9 — Additional file 9: Fig S6. Importance of random forest predictors for modelling AGB from backscatter HH and HV polarization, normalized difference backscatter index (NDBI) and texture measures from ALOS PALSAR as well as climatic water deficit (CWD). [file 13021_2020_151_MOESM9_ESM.tif]
