# Supplementary material for: Improving aboveground biomass maps of tropical dry forests by integrating LiDAR, ALOS PALSAR, climate and field data
Source: Carbon Balance Manag. 2020 Jul 29;15:15. doi: 10.1186/s13021-020-00151-6 (PMC7392681; doi:10.1186/s13021-020-00151-6)
Supplement: Supplementary file 5 — Additional file 5: Table S3. Regression parameters of the best model used to estimate aboveground biomass from LiDAR data. [file 13021_2020_151_MOESM5_ESM.docx]

Table S3. Regression parameters of the best model used to estimate aboveground biomass from LiDAR data

|  |  |  |
| --- | --- | --- |
| **Explanatory variables** | **β (Standard error)** | **R^2^** |
| Intercept | -0.81 (0.72) * | **0.87** |
| Elev AAD | 3.39 (0.61) ** |  |
| Elev MAD mode | -1.62 (0.28) ** |  |
| Percentage all returns above 4.00 | 0.14 (0.02) ** |  |
| * variables with p < 0.05; ** variables with p < 0.001. | |  |
